# Mortality, cardiovascular risk, and androgen deprivation therapy for prostate cancer: A systematic review with direct and network meta-analyses of randomized controlled trials and observational studies

**DOI:** 10.1097/MD.0000000000003873

**Published:** 2016-06-17

**Authors:** Lucie-Marie Scailteux, Florian Naudet, Quentin Alimi, Sébastien Vincendeau, Emmanuel Oger

**Affiliations:** a Pharmacovigilance, Pharmacoepidemiology and Drug Information Center, Rennes University Hospital, Rennes, France; b Clinical Investigation Center, INSERM 1414, Rennes University Hospital and University of Rennes 1, Rennes, France; c Urology Department, Rennes University Hospital, Rennes, France.

**Keywords:** androgen deprivation therapy, cardiovascular morbidity, cardiovascular mortality, network meta-analysis, prostate cancer

## Abstract

Androgen deprivation therapy (ADT) is a cornerstone therapy for advanced prostate cancer (PCa). We hypothesized that cardiovascular (CV) risk is different across the various ADT modalities to compare their effects on CV morbidity and mortality, and all-cause mortality in patients with PCa. To investigate more in depth potential CV risk heterogeneity focusing on coronary (main outcome) and cerebrovascular risk, CV, and overall mortality. We performed a Medline and Embase query, without language restriction, since 1950 up to July 2014. We included randomized controlled trials (RCTs) and observational studies providing that they compared at least 1 ADT modality to another one or to placebo and they gave data on CV event or all-cause mortality. Sixty-eight studies out of 3419 met our eligibility criteria. Eleven observational studies were analyzed. Direct meta-analyses showed that antiandrogen was associated with a 30% decrease risk for myocardial infarction (MI) compared to GnRH agonists (RR, 0.70 [0.54–0.91]); combined androgen blockade (CAB) was associated with a 10% increase risk for stroke when compared to antiandrogen (RR, 1.10 [1.02–1.19]). With regard to RCTs, 57 were included: direct meta-analyses suggested that CAB was associated with a 10% decrease of all-cause mortality when compared to GnRH agonist (RR, 0.90 [0.82–1.00]). Network analysis could only be performed for all-cause mortality and it remains difficult to disentangle benefit (positive impact on cancer survival) and risk (including CV risk). The impact of the ADT modalities on CV morbidity remains difficult to quantify and more detailed prospective collection is required. Registration: PROSPERO, CRD42014010598.

## Abstract 2

Supplemental Digital Content is available in the text

## 1 Introduction

Prostate cancer (PCa) is the most frequently diagnosed male cancer in the United States (US) and Europe.^[1]^ It is the third leading cause of cancer-related death, yet an increasing number of men are living longer with PCa. Androgen deprivation therapy (ADT) is considered a cornerstone treatment for advanced symptomatic metastatic disease yet it is also used alone or in association with radiotherapy (RT) to treat less advanced tumors.^[2]^

Although data suggested that ADT, when associated with RT, can improve survival, its impact on cardiovascular (CV) event and CV risk is still controversial. Past^[3]^ and more recent health-record-based studies^[4,5]^ have reported positive association. The US FDA has recognized the potential adverse cardiometabolic profile of ADT^[6]^ and has recommended that a patients’ CV risk be assessed prior to treatment.^[7,8]^

However, when focusing on type of ADT modality, results from observational studies suggested heterogeneity in the risk for myocardial infarction (MI) as well as for stroke.^[5,9,10]^ However, indication bias remains a challenge, in particular in observational studies, the prescribers taking into account the individual benefic/risk balance and notably the comorbidities of their patients to choose the most suitable ADT modality. Nevertheless, risk heterogeneity across the different ADT modalities (GnRH agonist or antagonist, antiandrogens [AA], etc.) is plausible and may be explained by their different pharmacologic actions.

In randomized controlled trials (RCTs), secondary safety outcomes such as MI, stroke, or CV death were less frequently evaluated than all-cause mortality, particularly in patients with comorbidities. Several direct meta-analyses did not detect a statistically significant difference between maximal androgen blockade and GnRH agonist monotherapy,^[11]^ ADT (predominantly GnRH agonist) and no treatment,^[12]^ GnRH agonist and GnRH antagonist in patients without CV disease and naïve of any cancer treatment.^[13]^ Insufficient power and classification bias on such safety secondary outcomes prevent a definitive conclusion.

Therefore, to investigate more in depth potential CV risk heterogeneity, we performed direct and network meta-analyses comparing ADT modalities within each other (i.e., GnRH agonist versus complete androgen blockade [CAB], AA vs CAB, etc.). We focused on coronary and cerebrovascular risk, CV, and overall mortality. We included observational studies and RCTs in all PCa stage patients because their different designs (patients’ selection, main outcome studied, comparability, follow-up duration, etc.) lead to different biases and their results are thought to be complementary, thus they should be summarized but we subgrouped them by type of studies.

## 2 Evidence acquisition

### 2.1 Eligibility criteria

We undertook this study in accordance with the MOOSE and PRISMA statement.^[14–16]^ We looked for RCTs and observational studies published up to July 28, 2014 without language restriction provided that they gave data on hormone sensitive PCa patients comparing 1 ADT modality to another or to either RT or total prostatectomy or placebo and that they considered MI, ischemic stroke, CV death, and all-cause mortality as primary or secondary (safety) outcomes.

Eligible ADT modalities, grouped in pharmacological classes each considered clinically homogeneous, were the following: GnRH agonists (buserelin, leuprorelin, goserelin, etc.), GnRH antagonists (degarelix, abarelix), antiandrogens (steroidal: cyproterone [CPT] or nonsteroidal: flutamide, bicalutamide, etc.), estrogen (diethylstilbestrol, polyestradiol phosphate, estradiol, etc.), and orchiectomy (OT). CAB was defined as an association of AA and GnRH agonist.

Because new drugs (abiraterone [ABIRA] or enzalutamide [ENZ]) were evaluated on top of ADT in castrate-resistant PCa patients, we decided to exclude those trials as our target population was hormone-sensitive PCa patients.

The primary outcome was MI. Secondary outcomes were ischemic stroke, CV death, and all-cause mortality. CV death included all patients who died by an ischemic process (coronary heart disease, ischemic heart disease, acute MI, stroke); we excluded death by congestive heart failure, arrhythmia, sudden cardiac death, deep vein thrombosis, and pulmonary or arterial embolism. For studies with insufficient detail on CV death, the outcome was extracted when it was reported (“cardiovascular death” or “cardiovascular mortality,” following the authors’ definition).

### 2.2 Search strategy

Literature search using Medline and Embase. We included MESH terms of all synonyms such as: PCa, prostatic neoplasm, targeted drug classes (gonadotropin releasing hormone agonist, luteinizing hormone releasing hormone agonist, etc.) and molecule name (flutamide, goserelin, etc.). The search formulated by LMS was reviewed by EO. For complete query see Appendix Text 1. We included grey literature such as letters and abstracts presented at relevant conference meeting. Title, abstracts, and full-text screening was performed in duplicate by LMS and QA. References list of obtained articles were hand searched. This review was registered in PROSPERO database (CRD42014010598).

### 2.3 Data extraction and study selection

LMS and QA independently extracted data from the selected studies into a standardized spreadsheet. Discrepancies were resolved by discussion until consensus was reached. When a publication was written in a language not fluently spoken by one of the 2 main reviewers, a translator did the extraction and the work was validated with an English-language extraction. The inclusion of data from multiple reports as separate studies (duplicate, overlapping, or companion studies) was allowed only when targeted outcomes were different. For observational studies pooling several ADT modalities, the author was contacted to obtain details on each ADT group. To avoid misestimating risk related to a specific ADT modality, studies not clearly defining drug exposure were excluded. When there were missing data on a specific outcome, we attempted to contact authors to obtain the relevant missing data. If data were not obtained, the study was discarded from the analysis on that specific outcome.

### 2.4 Data collection

The following variables were recorded: details of study (year, design, name or registration number, country, financial support, total number of participants, follow-up duration, type of analysis in RCT); details of participants (median age, previous PCa treatment, cancer stage [T score, metastasis]); regimens (class, drugs, dose, timing of administration, length of treatment, number treated); outcomes measure (number of events for each treatment modality).

### 2.5 Quality assessment

LMS and EO independently assessed study quality using the Joanna Briggs reviewer's manual^[17]^ for evaluating study biases using different tools for RCTs and for observational studies. Disagreements were resolved first by discussion and then by consulting a third author for arbitration.

### 2.6 Data analysis

Direct meta-analyses integrate the results of multiple independent studies addressing the same comparison. By extension, network meta-analyses allow inferences into the comparative effectiveness of those therapies that may or may not have been directly compared against each other, providing the network is connected.

For each outcome, adjusted risk estimates provided were chosen. If it was not given, we determined treatment effect along with 95% confidence interval (CI) from available raw data.

The estimate of overall effect (summary measure) was calculated with its 95% CI for each pair wise meta-analysis (head to head direct evidence) using random effects models separately for observational studies and RCTs through SAS macros.^[18]^ To meta-analyze studies including no event in at least one arm, we used the statistical methods described by Kuss.^[19]^ Statistical heterogeneity was documented with the *I*^2^ statistic (50–90%: may represent substantial heterogeneity) and investigated graphically by inspecting forest plots.^[20]^ We then considered the variability in participant factors among trials and trial factors.

A stratification analysis on T stage could not be performed owing to the fact that these data were not always available; however, all T stages were homogeneously represented across studies without overrepresentation of a stage in particular.

Network meta-analysis was performed for RCTs. We used the graph–theoretical method^[21]^ (see Appendix Text 2 for the script network meta-analysis). Results were reported in terms of OR and 95% CI. We used a design-based decomposition of Cochran Q for assessing the homogeneity in the whole network, the homogeneity within designs, and the homogeneity/consistency between designs. It allows also an assessment of the consistency assumption after detaching the effect of single designs. We used a net heat plot, a graphical tool for locating inconsistency.^[22]^

Analyses were run with R statistical package^[23]^ and the netmeta library.^[24]^

Publication bias was investigated graphically using funnel plots for each meta-analysis when there were at least 4 studies. Funnel plot asymmetry was tested using the rank correlation test when there were at least 10 studies.^[25]^

### 2.7 Role of the funding source

This study received no funding.

### 2.8 Ethical review

Ethical approval was not necessary considering we used already published studies.

## 3 Results

**Figure 1:**
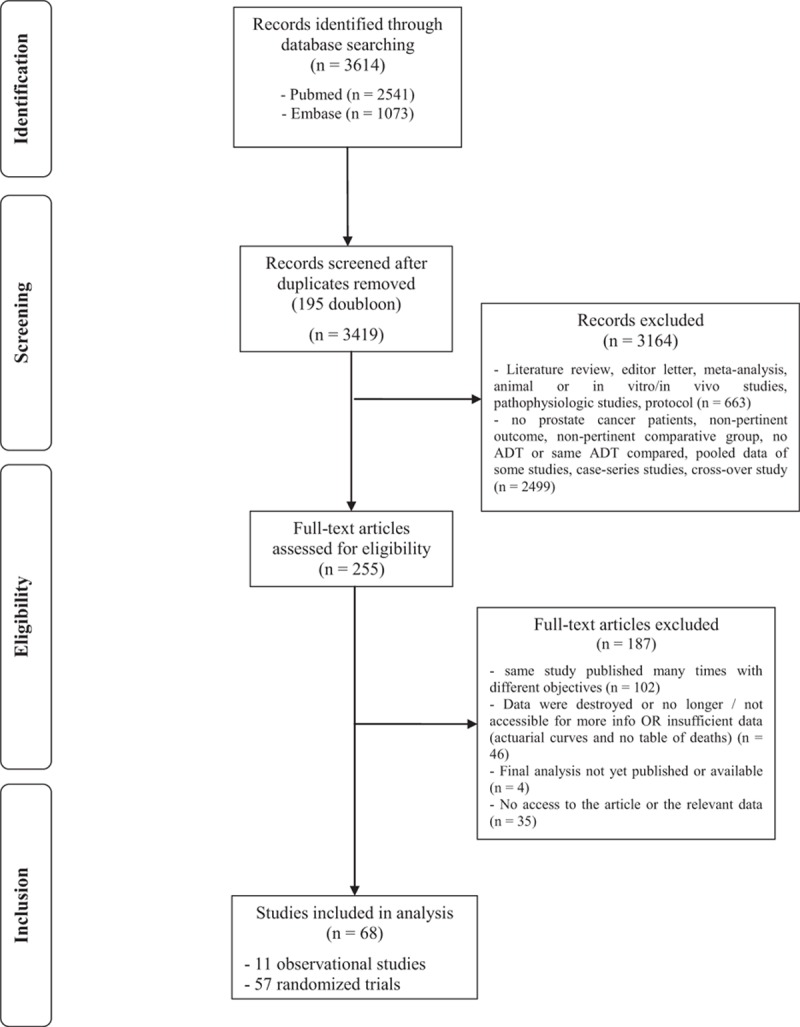
Summary of evidence search and selection.

Of the 3614 articles identified, 3419 left after deduplication (Fig. 1). After selection on the abstract then on full-text, 68 studies met our eligibility criteria: 11 observational studies and 57 RCTs. See Appendix eTable 1 which describes observational studies and Appendix eTable 2 which describes RCTs.

### 3.1 Observational studies

Studies pooling different ADT modalities were excluded, as well as CAPSURE or SEER^[26]^ studies which did not distinguish if LHRH agonist was alone or associated with AA. In the Jespersen et al study on the Danish Cancer Registry,^[10]^ the ICD-10 code BWHC covers LHRH agonist and AA modalities, and the isolated effect of LHRH agonists could not be assessed.

The 11 observational studies selected including 193,620 patients. Five studies^[5,9,27–29]^ gave data on CV morbidity (coronary and/or cerebrovascular risk) but only 1 study^[30]^ on CV death. The 6 studies given data on all-cause mortality (see Appendix Biblio) did not compare the same ADT modalities and could not be included in the meta-analysis.

Table 1 displays results from observational studies of the most frequently used ADT modalities. In synthesis, we observed an increased risk for stroke with CAB compared to AA (RR, 1.10 [1.02–1.19]); an increased risk for MI with GnRH agonist when compared to AA (RR, 1.43 [1.10–1.85]) and a consistent statistically nonsignificant association as regards stroke (RR, 1.22 [0.93–1.61]); thus AA appeared associated with a lower CV risk than GnRH alone or CAB. Appendix eTable 3 shows all comparisons with head to head direct comparison. When a relevant comparison (2 ADT compared in each other) was not available, we recalculated a relative risk from raw data. Data on CV death and all-cause mortality were sparse in observational studies.

**Table 1:**
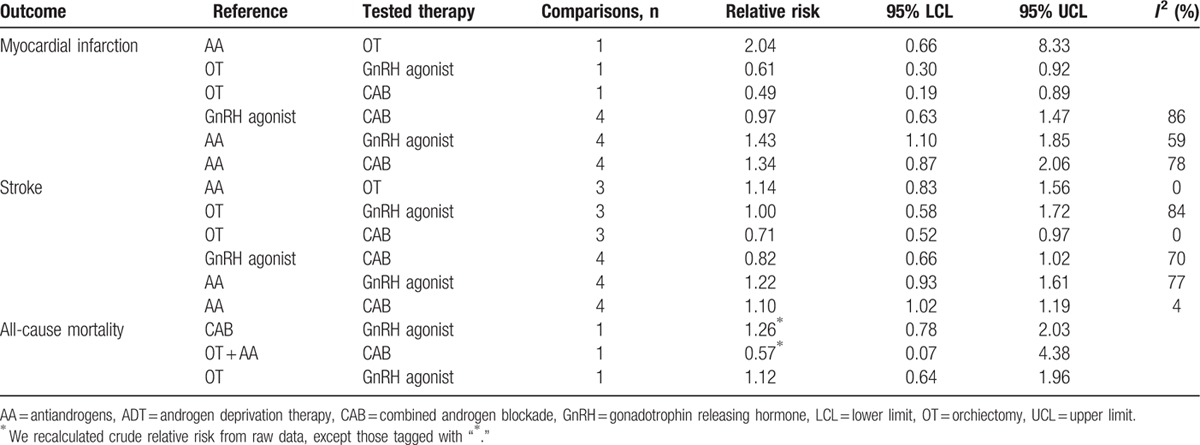
Result of the most frequently used ADT modalities from observational studies with direct meta-analyses.

See Appendix eTable 4 for quality assessment.

See Appendix eFigure 1 for funnel plot for publication bias. Of note, the limited number of studies (only 4 or 5 studies per head to head comparisons) hampered clearly ruling out publication bias.

### 3.2 Randomized controlled trials

Fifty-seven RCTs were included with 31,037 patients. Seven studies contained data on MI^[31–37]^ (101 events out of 2243 patients), 6 on stroke^[31–37]^ (35 events out of 2008 patients), 18 on CV death^[32–36,38–49]^ (1126 CV deaths out of 7787 patients), and 47 on all-cause mortality (see Appendix Biblio) (11,498 deaths out of 28,643 patients). Many ADT modalities were found and notably for CAB which required precise definition: short-term CAB corresponded to 3 or 4 months treatment; long-term CAB to only 6 to 8 months treatment; continuous CAB was a very long term (>1 year or permanent) treatment contrary to intermittent CAB which was also given on a very long term but episodically often because of progression or relapse of PCa disease.

Two publications were used to extract data on different outcomes in each, respectively the RTOG study 92-02^[44,50]^ and the degarelix study.^[37,51]^ As regards the 6 RCTs comparing GnRH antagonist to GnRH agonist included in Albertsen meta-analysis,^[13]^ only 1 trial fulfilled our eligibility criteria (CS21^[37,51]^); for CS28, CS30, and CS31, no data concerning our outcomes were available; CS35 and CS37 were currently not published: some results are available on the website www.clinicaltrials.gov but no data on CV risk or mortality (CV or overall) are displayed for CS35, and no results nor publication are available for CS37. We did not use estimates reported by Albertsen because eligibility was supported by access to individual data studies published or not.

Table 2 displays results of the most frequently used ADT modalities from RCT. Appendix eTable 5 shows all comparisons with head-to-head direct comparison focusing on trials comparing active therapies. When a treatment effect for our chosen outcomes was available, we recalculated a relative risk from raw data. MI, stroke, and CV death were rarely reported and direct meta-analyses included only 2 studies; no difference was detected. All-cause mortality was reported as a main outcome or described in the safety data. As shown in Table 2, GnRH agonist was associated with a slight decrease in risk of all-cause mortality when compared to OT (OR, 0.93 [0.86–1.00]), as continuous CAB when compared to GnRH agonist (OR, 0.90 [0.82–1.00]).

**Table 2:**
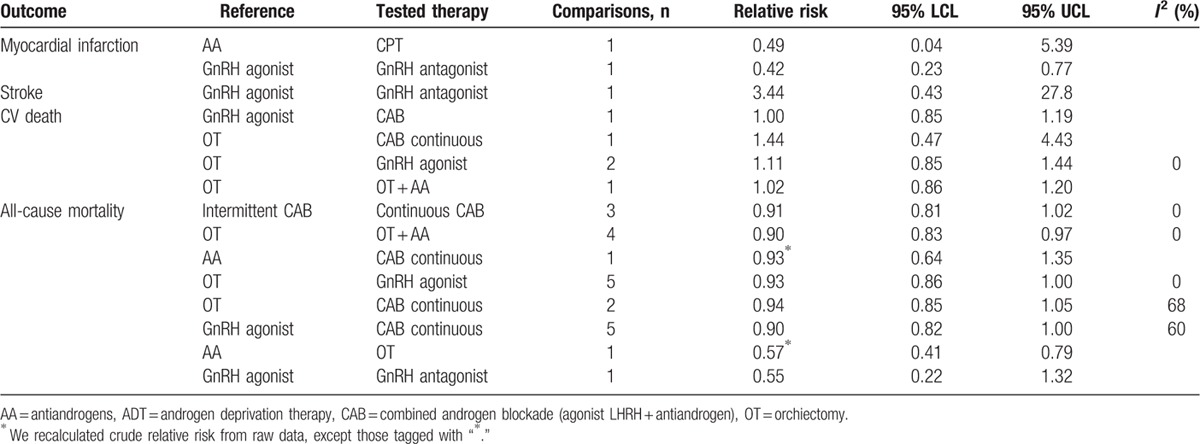
Results of the most frequently used ADT modalities from randomized controlled trials with direct meta-analyses.

See Appendix eFigure 1 for the funnel plots for publication bias. Once again, the limited number of RCTs hampered clearly ruling out publication bias. See also Appendix eTable 6 for quality assessment.

### 3.3 Network meta-analysis

A closed network could only be drawn for all-cause mortality because there were too scarce data for MI, stroke, and CV death outcomes.

Table 3 shows comparisons between the most frequently used ADT modalities. Appendix eTable 7 shows all comparisons between the different ADT modalities (see also Appendix eFigure 2 when using LHRH agonist as the reference). Appendix eFigure 3 shows the network model for all-cause mortality: there were a large number of ADT modalities and few RCTs comparing them.

**Table 3:**
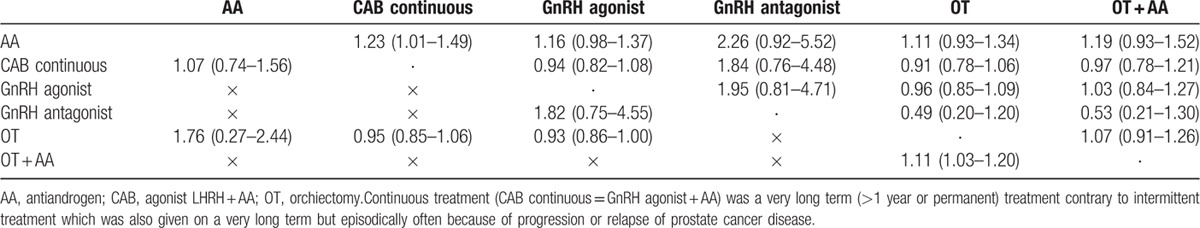
Analysis from randomized controlled trials: the upper right side concern the indirect comparisons (network) with OR (95% CL) for all-cause mortality (the reference treatment appears in the column), and the lower left side concern the direct analysis (the reference treatment appears in the line).

In synthesis, we observed that AA had a 23% increased risk for all-cause mortality compared to continuous CAB (RR, 1.23 [1.01–1.49]) and a somewhat similar increase though not statistically significant when compared to the other main ADT modalities. Otherwise, we did not detect a substantial difference in overall survival between GnRH agonist, GnRH antagonist, continuous CAB, and OT.

Inconsistency and heterogeneity were identified across the network; inconsistency could have influence comparison “AA–OT” or the “long-term CAB–short-term CAB” (Appendix eTable 8).

## 4 Discussion

### 4.1 Main findings

Our results support the hypothesis that the various ADT modalities have a different impact on CV risk. Focusing on MI and stroke, we observed through a comprehensive quantitative synthesis (direct meta-analysis) of observational studies that CAB differed from AA which differed from GnRH agonists.

### 4.2 Strengths

Our systematic review encompassed a large panel of observational studies and RCTs.

We excluded studies with pooled ADT modalities in their analyses or without clear definition of CV outcomes to avoid including studies with potential misclassification either on drug exposure or outcome that would have blurred relevant data from other studies more suited to our specific purpose.

Finally, indirect network meta-analysis gave the opportunity to estimate treatment effect between 2 ADT modalities without head-to-head data available.

### 4.3 Limits

Direct meta-analyses included very few studies (at most 5 for all-cause mortality). In addition, they suffered from substantial heterogeneity which could be related to population characteristics and methodological parameters. Analysis of publication bias could not be ruled out as funnel plots included only 4 or 5 studies. Network meta-analysis suffered from inconsistency in some comparisons. Eventually, no firm conclusion could be drawn from these data.

Data on MI, stroke, and CV death were limited especially in RCTs. As regards all-cause mortality, it remains difficult to disentangle benefit with better survival through a positive impact on cancer progression and risk including CV risk. Cancer staging such as presence of metastasis and CV history are major issues and can induce a shorter survival duration compared with nonmetastatic patients who could have time to develop CV disorders and in whom CV death can be anticipated. The negative prognostic impact of severe comorbidity could also be due to cancer therapy adapted to comorbidity making it difficult to discern whether worse survival is due to comorbidity or less efficacious treatment. This is notably claimed by a study on the importance of comorbidity in cancer patients.^[52]^ RCT included in our meta-analysis were rarely stratified on CV comorbidity including coronary heart disease or cerebrovascular disease, and we did not explore the risk of CV death nor all-cause mortality across CV comorbidity.

### 4.4 Comparison to other studies

Previous meta-analyses have been published but did not precisely address our hypothesis because of an analysis that pooled several ADT modalities,^[11,12,53,54]^ a no treatment comparison group,^[54,55]^ restrictive criteria, or different objectives. The first meta-analysis^[11]^ including 27 trials which focused on metastatic (88%) and locally advanced (12%) PCa patients concluded that maximal androgen blockade [MAB] (OT + AA or CAB or OT + CPT) improved the 5-year survival by about 2% or 3% compared to androgen suppression alone [AS] (OT or LHRH agonist). The second meta-analysis^[12]^ analyzed data from 8 studies enrolling nonmetastatic and nonhormone-refractory PCa patients and did not detect any evidence that immediate ADT (pooling several ADT modalities) increased CV death compared to no immediate ADT. The third study^[53]^ reanalyzed data from the previous meta-analysis^[12]^ and showed a nonsignificant association between CV death and ADT use. The fourth meta-analysis^[55]^ included 8 observational studies assessing the risk of fatal and nonfatal CV event with different ADT modalities compared to no treatment, irrespective of PCa stage. A fifth and more recent meta-analysis identified 6 population-based observational studies comparing ADT modality versus watchful waiting or active surveillance.^[54]^ Some studies used data from SEER database^[26]^ or some national cancer registries^[10]^ where extracting codes included many modalities, such as LHRH agonist and AA without clearly distinguishing them. Results indicated that LHRH agonists were associated with an increased risk for stroke and MI (fatal or not) and that AA were associated with an increased risk for any nonfatal CV disease compared to no treatment. We excluded these studies to avoid any mixing between ADT modalities. Nevertheless, we also found an increased risk of MI and stroke with LHRH agonist versus no endocrine treatment, as well as OT when compared with no endocrine treatment.

Another meta-analysis^[13]^ focused on 6 trials comparing LHRH agonist to LHRH antagonist in metastatic and nonmetastatic, locally or not advanced PCa patients, naïve of ADT treatment. Safety data including CV morbidity, CV mortality, and all-cause mortality were scarce due to the small follow-up duration (12 months); in 2 trials, data were not available or published. Results indicated, in patients with CV history, a decrease in cardiac event with LHRH antagonist patients compared to LHRH agonist. The last meta-analysis focused on 8 RCTs comparing intermittent androgen deprivation to continuous androgen deprivation and did not detect any difference in overall survival.^[56]^

From a pharmacological point of view, a differential impact of the various ADT modalities on CV risk might be explained. Studies have established that ADT could increase weight gain, body fat percentage, triglycerides rate, and decrease lean body mass, and insulin sensitivity.^[57–59]^ The link with diabetes, metabolic syndrome, and ADT is claimed by some authors^[58,59]^; those metabolic changes may increase CV risk but the underlying mechanism remains unclear. ADT suppress androgen activity by central or peripheral mechanisms: by decreasing testicular and/or extra gonadal androgen production with GnRH agonist and antagonist or by blocking androgen receptor activation using AA. GnRH receptors are synthesized in several extrapituitary tissues as well as the reproductive system,^[60]^ bladder,^[61]^ or heart.^[62]^ Dong et al^[63]^ observed an impact of the GnRH agonist on cardiomyocytes contractile function in mice. Other authors^[64,65]^ suggested, through studies on human mononuclear cells, that GnRH receptors located on T-lymphocytes could indirectly explain a modification of the stability of the atheromatous plaques due to their activation and proliferation after administration of GnRH agonist (T-lymphocytes are the main immune cells infiltrating the atheromatous plaques). This hypothesis could explain the increase of cerebrovascular and coronary heart diseases observed with GnRH agonists.

## 5 Conclusion

Our results support the hypothesis that the various ADT modalities have different impact as regards CV risk.

However, we should be cautious and consider that the question is currently not totally resolved. RCT does not seem adapted to this issue and we are currently conducting a large nationwide population-based study (ADTCR) using the French medico-administrative database.

## Supplementary Material

Supplemental Digital Content
