# Supplementary material for: Mortality, cardiovascular risk, and androgen deprivation therapy for prostate cancer: A systematic review with direct and network meta-analyses of randomized controlled trials and observational studies
Source: Medicine (Baltimore). 2016 Jun 17;95(24):e3873. doi: 10.1097/MD.0000000000003873 (PMC4998460; doi:10.1097/MD.0000000000003873)
Supplement: Supplemental Digital Content [file medi-95-e3873-s001.doc]

**Appendix biblio. References of studies describing all cause-mortality.**

- **Observational studies:**

- Bittner N, Merrick GS, Galbreath RW, et al. Primary Causes of Death After Permanent Prostate Brachytherapy. Int J Radiat Oncol Biol Phys. 2008;72(2):433-440.

- D’Amico AV, Loffredo M, Renshaw AA, Loffredo B, Chen M-H. Six-month androgen suppression plus radiation therapy compared with radiation therapy alone for men with prostate cancer and a rapidly increasing pretreatment prostate-specific antigen level. J Clin Oncol. 2006;24(25):4190-4195.

- Koutsilieris M, Faure N, Tolis G, Laroche B, Robert G, Ackman CF. Objective response and disease outcome in 59 patients with stage D2 prostatic cancer treated with either Buserelin or orchiectomy. Disease aggressivity and its association with response and outcome. Urology. 1986;27(3):221-228.

- Matsumoto K, Hagiwara M, Tanaka N, et al. Survival following primary androgen deprivation therapy for localized intermediate- or high-risk prostate cancer: comparison with the life expectancy of the age-matched normal population. Med Oncol Northwood Lond Engl. 2014;31(6):979.

- Nanda A, Chen M-H, Moran BJ, Braccioforte MH, D’Amico AV. Cardiovascular comorbidity and mortality in men with prostate cancer treated with brachytherapy-based radiation with or without hormonal therapy. Int J Radiat Oncol Biol Phys. 2013;85(5):e209-e215.

- Parekh A, Chen M-H, D’Amico AV, et al. Identification of comorbidities that place men at highest risk of death from androgen deprivation therapy before brachytherapy for prostate cancer. Brachytherapy. 2013;12(5):415-421.

- **Randomized clinical trials:**

- Akaza H, Hinotsu S, Usami M, et al. Combined androgen blockade with bicalutamide for advanced prostate cancer: Long-term follow-up of a phase 3, double-blind, randomized study for survival. Cancer. 2009;115(15):3437-3445.

- Akaza H, Homma Y, Okada K, et al. A prospective and randomized study of primary hormonal therapy for patients with localized or locally advanced prostate cancer unsuitable for radical prostatectomy: results of the 5-year follow-up. BJU Int. 2003;91(1):33-36.

- Anderson J, Al-Ali G, Wirth M, et al. Degarelix versus goserelin (+ antiandrogen flare protection) in the relief of lower urinary tract symptoms secondary to prostate cancer: results from a phase IIIb study (NCT00831233). Urol Int. 2013;90(3):321-328.

- Ansari MS, Gupta NP, Hemal AK, Dogra PN, Seth A. Combined androgen blockade in the management of advanced prostate cancer: a sensible or ostensible approach. Int J Urol Off J Jpn Urol Assoc. 2004;11(12):1092-1096.

- Armstrong JG, Gillham CM, Dunne MT, et al. A randomized trial (Irish clinical oncology research group 97-01) comparing short versus protracted neoadjuvant hormonal therapy before radiotherapy for localized prostate cancer. Int J Radiat Oncol Biol Phys. 2011;81(1):35-45.

- Bales GT, Chodak GW. A controlled trial of bicalutamide versus castration in patients with advanced prostate cancer. Urology. 1996;47(1A Suppl):38-43; discussion 48-53.

- Boccardo F, Barichello M, Battaglia M, et al. Bicalutamide monotherapy versus flutamide plus goserelin in prostate cancer: updated results of a multicentric trial. Eur Urol. 2002;42(5):481-490.

- Boccardo F, Pace M, Rubagotti A, et al. Goserelin acetate with or without flutamide in the treatment of patients with locally advanced or metastatic prostate cancer. Eur J Cancer. 1993;29(8):1088-1093.

- Bolla M, de Reijke TM, Van Tienhoven G, et al. Duration of androgen suppression in the treatment of prostate cancer. N Engl J Med. 2009;360(24):2516-2527.

- Bolla M, Van Tienhoven G, Warde P, et al. External irradiation with or without long-term androgen suppression for prostate cancer with high metastatic risk: 10-year results of an EORTC randomised study. Lancet Oncol. 2010;11(11):1066-1073.

- Botto H, Richard F, Mathieu F, Camey M. Decapeptyl in the treatment of advanced prostatic cancer: comparative study with pulpectomy. Prog Clin Biol Res. 1989;303:53-60.

- Brisset JM, Boccon-Gibod L, Botto H, et al. Anandron (RU 23908) associated to surgical castration in previously untreated stage D prostate cancer: a multicenter comparative study of two doses of the drug and of a placebo. Prog Clin Biol Res. 1987;243 A:411-422.

- Bruun E, Frimodt-Møller C. The effect of Buserelin versus conventional antiandrogenic treatment in patients with T2-4NXM1 prostatic cancer. A prospective, randomized multicentre phase III trial. The “Danish Buserelin Study Group.” Scand J Urol Nephrol. 1996;30(4):291-297.

- Burns-Cox N, Basketter V, Higgins B, Holmes S. Prospective randomised trial comparing diethylstilboestrol and flutamide in the treatment of hormone relapsed prostate cancer. Int J Urol Off J Jpn Urol Assoc. 2002;9(8):431-434.

- Calais da Silva FEC, Bono AV, Whelan P, et al. Intermittent androgen deprivation for locally advanced and metastatic prostate cancer: results from a randomised phase 3 study of the South European Uroncological Group. Eur Urol. 2009;55(6):1269-1277.

- Citrin DL, Resnick MI, Guinan P, et al. A comparison of Zoladex and DES in the treatment of advanced prostate cancer: results of a randomized, multicenter trial. The Prostate. 1991;18(2):139-146.

- Crawford ED, Eisenberger MA, McLeod DG, et al. A controlled trial of leuprolide with and without flutamide in prostatic carcinoma. N Engl J Med. 1989;321(7):419-424.

- D’Amico AV, Chen M-H, Renshaw AA, Loffredo M, Kantoff PW. Androgen suppression and radiation vs radiation alone for prostate cancer: a randomized trial. JAMA. 2008;299(3):289-295.

- De Voogt HJ, Studer U, Schroder FH, Klijn JG, De Pauw M, Sylvester R. Maximum androgen blockade using LHRH agonist buserelin in combination with short-term (two weeks) or long-term (continuous) cyproterone acetate is not superior to standard androgen deprivation in the treatment of advanced prostate cancer. Final analysis of EORTC GU group trial 30843. Eur Urol. 1998;33(2):152-158.

- Denham JW, Steigler A, Lamb DS, et al. Short-term neoadjuvant androgen deprivation and radiotherapy for locally advanced prostate cancer: 10-year data from the TROG 96.01 randomised trial. Lancet Oncol. 2011;12(5):451-459.

- Denis LJ, Keuppens F, Smith PH, et al. Maximal androgen blockade: Final analysis of EORTC phase III trial 30853. Eur Urol. 1998;33(2):144-151.

- Dijkman GA, Janknegt RA, De Reijke TM, Debruyne FM. Long-term efficacy and safety of nilutamide plus castration in advanced prostate cancer, and the significance of early prostate specific antigen normalization. International Anandron Study Group. J Urol. 1997;158(1):160-163.

- Eisenberger MA, Blumenstein BA, Crawford ED, et al. Bilateral orchiectomy with or without flutamide for metastatic prostate cancer. N Engl J Med. 1998;339(15):1036-1042.

- Horwitz EM, Bae K, Hanks GE, et al. Ten-year follow-up of radiation therapy oncology group protocol 92-02: A phase III trial of the duration of elective androgen deprivation in locally advanced prostate cancer. J Clin Oncol. 2008;26(15):2497-2504.

- Hussain M, Tangen CM, Berry DL, et al. Intermittent versus continuous androgen deprivation in prostate cancer. N Engl J Med. 2013;368(14):1314-1325.

- Irani J, Celhay O, Hubert J, et al. Continuous versus six months a year maximal androgen blockade in the management of prostate cancer: a randomised study. Eur Urol. 2008;54(2):382-391.

- Iversen P, McLeod DG, See WA, et al. Antiandrogen monotherapy in patients with localized or locally advanced prostate cancer: final results from the bicalutamide Early Prostate Cancer programme at a median follow-up of 9.7 years. BJU Int. 2010;105(8):1074-1081. doi:10.1111/j.1464-410X.2010.09319.x.

- Iversen P, Rasmussen F, Klarskov P, Christensen IJ. Long-term results of Danish Prostatic Cancer Group trial 86. Goserelin acetate plus flutamide versus orchiectomy in advanced prostate cancer. Cancer. 1993;72(12 Suppl):3851-3854.

- Jones CU, Hunt D, McGowan DG, et al. Radiotherapy and short-term androgen deprivation for localized prostate cancer. N Engl J Med. 2011;365(2):107-118.

- Kaisary AV, Tyrrell CJ, Peeling WB, Griffiths K. Comparison of LHRH analogue (Zoladex) with orchiectomy in patients with metastatic prostatic carcinoma. Br J Urol. 1991;67(5):502-508.

- Kotake T, Usami M, Akaza H, et al. Goserelin acetate with or without antiandrogen or estrogen in the treatment of patients with advanced prostate cancer: A multicenter, randomized, controlled trial in Japan. Jpn J Clin Oncol. 1999;29(11):562-570.

- Lukkarinen O, Kontturi M. Comparison of a long-acting LHRH agonist and polyoestradiol phosphate in the treatment of advanced prostatic carcinoma. An open prospective, randomized multicentre study. Scand J Urol Nephrol. 1994;28(2):171-178.

- Manikandan R, Srirangam SJ, Pearson E, Brown SCW, O’Reilly P, Collins GN. Diethylstilboestrol versus bicalutamide in hormone refractory prostate carcinoma: a prospective randomized trial. Urol Int. 2005;75(3):217-221.

- Mottet N, Van Damme J, Loulidi S, et al. Intermittent hormonal therapy in the treatment of metastatic prostate cancer: a randomized trial. BJU Int. 2012;110(9):1262-1269.

- Navratil H. Double-blind study of Anandron versus placebo in stage D2 prostate cancer patients receiving buserelin. Results on 49 cases from a multicentre study. Prog Clin Biol Res. 1987;243A:401-410.

- Organ M, Wood L, Wilke D, et al. Intermittent LHRH therapy in the management of castrate-resistant prostate cancer (CRPCa): results of a multi-institutional randomized prospective clinical trial. Am J Clin Oncol. 2013;36(6):601-605.

- Ostri P, Bonnesen T, Nilsson T, Frimodt-Møller C. Treatment of symptomatic metastatic prostatic cancer with cyproterone acetate versus orchiectomy: a prospective randomized trial. Urol Int. 1991;46(2):167-171.

- Parmar H, Phillips RH, Lightman SL, Edwards L. How would you like to have an orchidectomy for advanced prostatic cancer? Am J Clin Oncol. 1988;11 Suppl 2:S160-S168.

- Pavone-Macaluso M, de Voogt HJ, Viggiano G, et al. Comparison of diethylstilbestrol, cyproterone acetate and medroxyprogesterone acetate in the treatment of advanced prostatic cancer: Final analysis of a randomized phase III trial of the European Organization for Research on Treatment of Cancer Urological Group. J Urol. 1986;136(3):624-631.

- Roach III M, Bae K, Speight J, et al. Short-term neoadjuvant androgen deprivation therapy and external-beam radiotherapy for locally advanced prostate cancer: Long-term results of RTOG 8610. J Clin Oncol. 2008;26(4):585-591.

- Robinson MR, Smith PH, Richards B, Newling DW, de Pauw M, Sylvester R. The final analysis of the EORTC Genito-Urinary Tract Cancer Co-Operative Group phase III clinical trial (protocol 30805) comparing orchidectomy, orchidectomy plus cyproterone acetate and low dose stilboestrol in the management of metastatic carcinoma of the prostate. Eur Urol. 1995;28(4):273-283.

- Schröder FH, Whelan P, De Reijke TM, et al. Metastatic prostate cancer treated by Flutamide versus Cyproterone acetate: Final analysis of the “European Organization for Research and Treatment of Cancer” (EORTC) protocol 30892. Eur Urol. 2004;45(4):457-464.

- Sharifi R, Lee M, Ojeda L, Ray P, Stobnicki M, Guinan P. Comparison of leuprolide and diethylstilbestrol for stage D2 adenocarcinoma of prostate. Urology. 1985;26(2):117-124.

- Thorpe SC, Azmatullah S, Fellows GJ, Gingell JC, O’Boyle PJ. A prospective, randomised study to compare goserelin acetate (Zoladex(®)) versus cyproterone acetate (Cyprostat(®)) versus a combination of the two in the treatment of metastatic prostatic carcinoma. Eur Urol. 1996;29(1):47-54.

- Tyrrell CJ, Altwein JE, Klippel F, et al. Comparison of an LH-RH analogue (Goeserelin acetate, “Zoladex”) with combined androgen blockade in advanced prostate cancer: final survival results of an international multicentre randomized-trial. International Prostate Cancer Study Group. Eur Urol. 2000;37(2):205-211.

- Waymont B, Lynch TH, Dunn JA, et al. Phase III randomised study of zoladex versus stilboestrol in the treatment of advanced prostate cancer. Br J Urol. 1992;69(6):614-620.

- Wirth MP, Weissbach L, Marx F-J, et al. Prospective randomized trial comparing flutamide as adjuvant treatment versus observation after radical prostatectomy for locally advanced, lymph node-negative prostate cancer. Eur Urol. 2004;45(3):267-270; discussion 270.

**Appendix eFigure 1. Funnel plot for publication bias.**

Funnel plot for stroke in observational studies

Comparison “GnRH agonist versus CAB” Comparison “GnRH agonist versus AA” Comparison “AA versus CAB”

Funnel plot for myocardial infarction in observational studies

Comparison “GnRH agonist versus CAB” Comparison “GnRH agonist versus AA” Comparison “AA versus CAB”

Comparison “ No endocrine treatment versus GnRH agonist” Comparison “ No endocrine treatment versus AA” Comparison “ No endocrine treatment versus CAB”

Funnel plot for overall death in RCTs

Comparison “placebo versus CAB short term” Comparison “placebo versus GnRH agonist” Comparison “placebo versus AA”

Comparison “OT versus OT + AA” Comparison “OT versus GnRH agonist” Comparison “Estrogen versus GnRH agonist”

Comparison “GnRH agonist versus CAB continuous”

AA: antiandrogen

CAB: combined androgen blockade (GnRH agonist + AA)

OT: orchidectomy

**Appendix eFigure 2.** Estimate from network meta-analysis for overall death (LHRH agonist is the reference).

AA : antiandrogen – CAB : agonist LHRH + AA – ABIRA : abiraterone – ENZ: enzalutamide – OT: orchiectomy – CPT : cyproterone acetate – CMD : chlormadinone – DES: diethylstilbestrol – LHRH = GnRH.

**Appendix** **eFigure 3. Treatment network of overall death for all studies.**

Line’s thickness is proportional to the number of studies comparing the corresponding ADT modalities.

ABIRA : abiraterone – AA: antiandrogen – BT: brachytherapy – CAB: Combined androgen blockade = GnRH agonist + antiandrogen – CMD : chlormadinone – CPT: cyproterone – DES: diethylstilbestrol – ENZ : enzalutamide – OT: orchidectomy – PEP: polyestradiol phosphate – RT: radiotherapy.

- Short term CAB corresponded to 3 or 4 months treatment.

- Long term CAB to only 6 to 8 months treatment.

- Continuous treatment was a very long term (> 1 year or permanent) treatment contrary to intermittent treatment which was also given on a very long term but episodically often because of progression or relapse of prostate cancer disease.

- CMD long term: at least 24 months.

- CMD short term: 8 weeks.

- DES short term: 8 weeks.

Appendix eTable 1. Extracted observational studies

| First author | Journal | Publication year | Country | Patients  N | Median age (years) | Participants naïve of treatment | T-score Metastasis | Prostate cancer risk group most frequently observed | CV history | Drugs compared | Follow-up duration  (years) | Data provided on outcome | | | |
| --- | --- | --- | --- | --- | --- | --- | --- | --- | --- | --- | --- | --- | --- | --- | --- |
|  |  |  |  |  |  |  |  |  |  |  |  | MI | Stroke | CV death | Overall death |
| D'Amico | J Clin Oncol | 2006 | USA | 241 | 73 | Yes | T1c-T3 | Intermediate | NA | RT RT + CAB | 4.6 | No | No | No | Yes |
| Robinson | International Journal of Cancer | 2012 | Sweden | 39 051 | 75% <75 | Yes | T1-T4 M1 | Low or intermediate (69%) | MI (12%) Stroke (7.5%) | No treatment GnRH agonist AA, CAB | 1.9 | Yes | Yes | No | No |
| Bittner | Int J Radiat Oncol Biol Phys | 2008 | USA | 1 354 | 66 | Yes | T1-T3 | Low or intermediate (82%) | Hypertension (48%) Diabetes (48%) | BT BT + CAB ≤ 6 mo BT + CAB > 6 mo | 5.4 | No | No | Yes | Yes |
| Nanda | Int J Radiat Oncol Biol Phys | 2012 | USA | 5 077 | 69 | Yes | T1-T3, N0, M0 | Low | Hypertension, diabetes or dyslipidaemia (43%) | BT  BT + CAB | 4.8 | No | No | No | Yes |
| Matsumoto | Medical Oncology | 2014 | Japan | 410 | 76 |  | T1-T3, N0, M0 | High (59.5%) | not given | GnRH agonist  CAB | 6.0 | No | No | No | Yes |
| Van Hemelrijck | European Urologia | 2012 | Sweden | 76 600 | 90 % > 65 | Yes | M1 (40% of ADT treated patients) | High | (33% to 56%) | Watching GnRH agonist AA, CAB, OT | 4.0 | Yes | Yes | No | No |
| Koutsilieris | Urology | 1986 | Canada | 59 | NA | Yes | D2 | High | not given | OT GnRH agonist | 3.0 | No | No | No | Yes |
| Parekh | Brachytherapy | 2013 | USA | 5972 | 72 | Yes | T1-T3 | Low or intermediate (81%) | MI or coronary HF (8.2%) Diabetes (7.7%) Hypertension and hyperchol. (29%) | BT BT + GnRH agonist | 4.0 | No | No | No | Yes |
| Keating | J Natl Cancer Inst | 2010 | USA | 37443 | 66.9 | Yes | Local or regional, M0 | NA | Overall (29%) | No treatment  GnRH agonist AA, CAB, OT | 2.6 | Yes | Yes | No | No |
| Azoulay | European Urology | 2011 | Canada | 22310 | 72.3 | Yes | M0 | NA | MI (0.9%), HF (6%)  Diabetes (9.9%) Hypertension (37.2%), | No treatment  GnRH agonist  AA, CAB, OT | 3.9 | No | Yes | No | No |
| Martín-Merino | Drug safety | 2011 | Europe | 5103 | 72 | Yes | Mostly M0 | NA | IHD (47%)  Stroke (18%)  Diabetes (17%)  Hyperchol. (22%)  Hypertension (51%) | no treatment  GnRH agonist  AA, CAB, OT | 7.0 | Yes | Yes | No | No |

AA: antiandrogen – BT: brachytherapy – CAB: Combined androgen blockade = GnRH agonist + antiandrogen – OT: orchidectomy – RT: radiotherapy.

M1 = metastatic disease – NA: not available.

Bibliography

- Azoulay L, Yin H, Benayoun S, Renoux C, Boivin J-F, Suissa S. Androgen-deprivation therapy and the risk of stroke in patients with prostate cancer. Eur Urol. 2011;60(6):1244-1250.

- Bittner N, Merrick GS, Galbreath RW, et al. Primary Causes of Death After Permanent Prostate Brachytherapy. Int J Radiat Oncol Biol Phys. 2008;72(2):433-440.

- D’Amico AV, Loffredo M, Renshaw AA, Loffredo B, Chen M-H. Six-month androgen suppression plus radiation therapy compared with radiation therapy alone for men with prostate cancer and a rapidly increasing pretreatment prostate-specific antigen level. J Clin Oncol. 2006;24(25):4190-4195.

- Keating NL, O’Malley AJ, Freedland SJ, Smith MR. Diabetes and cardiovascular disease during androgen deprivation therapy: Observational study of veterans with prostate cancer. J Natl Cancer Inst. 2010;102(1):39-46.

- Koutsilieris M, Faure N, Tolis G, Laroche B, Robert G, Ackman CF. Objective response and disease outcome in 59 patients with stage D2 prostatic cancer treated with either Buserelin or orchiectomy. Disease aggressivity and its association with response and outcome. Urology. 1986;27(3):221-228.

- Martín-Merino E, Johansson S, Morris T, García Rodríguez LA. Androgen deprivation therapy and the risk of coronary heart disease and heart failure in patients with prostate cancer: A nested case-control study in UK primary care. Drug Saf. 2011;34(11):1061-1077.

- Matsumoto K, Hagiwara M, Tanaka N, et al. Survival following primary androgen deprivation therapy for localized intermediate- or high-risk prostate cancer: comparison with the life expectancy of the age-matched normal population. Med Oncol Northwood Lond Engl. 2014;31(6):979.

- Nanda A, Chen M-H, Moran BJ, Braccioforte MH, D’Amico AV. Cardiovascular comorbidity and mortality in men with prostate cancer treated with brachytherapy-based radiation with or without hormonal therapy. Int J Radiat Oncol Biol Phys. 2013;85(5):e209-e215.

- Parekh A, Chen M-H, D’Amico AV, et al. Identification of comorbidities that place men at highest risk of death from androgen deprivation therapy before brachytherapy for prostate cancer. Brachytherapy. 2013;12(5):415-421..

- Robinson D, Garmo H, Lindahl B, et al. Ischemic heart disease and stroke before and during endocrine treatment for prostate cancer in PCBaSe Sweden. Int J Cancer. 2012;130(2):478-487.

- Van Hemelrijck M, Garmo H, Holmberg L, et al. Absolute and relative risk of cardiovascular disease in men with prostate cancer: Results from the population-based PCBaSe Sweden. J Clin Oncol. 2010;28(21):3448-3456.

**Online-only eTable2**: **Extracted randomized controlled trials**

| First author | Journal | Publication  year | Country | Patients  N | Median age (years) | Participants naïve of treatment | T-score Metastasis | CV history | Drugs compared | Follow-up duration  (years) | Data provided on outcome | | | |
| --- | --- | --- | --- | --- | --- | --- | --- | --- | --- | --- | --- | --- | --- | --- |
|  |  |  |  |  |  |  |  |  |  |  | MI | Stroke | CV death | Overall death |
| D'Amico | JAMA | 2008 | USA | 206 | 75 | Yes | T1- T2, N0, M0 | Moderate or severe comorbidity (25%) | RT RT + short term CAB | 7.6 y | No | No | No | Yes |
| Hussain | NEJM | 2013 | USA, Canada | 1 535 | 70 | Drugs (≈28%) RT (≈29%), PT (≈20%) | M1 | NA | Intermittent CAB Continuous CAB | 9.8 y | No | No | No | Yes |
| Mottet | BJUI | 2012 | Europe | 173 | 69 | No (CAB) | M1 | NA | Intermittent CAB Continuous CAB | 3.7 y | No | No | No | Yes |
| Jones | NEJM | 2011 | USA, Canada | 1979 | 70 | No | T1-T2, Nx, M0 | NA | RT RT + short term CAB | 9.2 y | No | No | No | Yes |
| Denham | Lancet Oncology | 2011 | Australia, New Zealand | 802 | 70 | Yes | T2-T4, M0 | NA | RT alone RT + CAB 3 mo. RT + CAB 6 mo. | 10.6 y | No | No | Yes | Yes |
| Bolla | Lancet Oncology | 2010 | International | 415 | 70 | Yes | T1-T4, M0 | NA | RT RT + GnRH agonist | 9.1 y | No | No | Yes | Yes |
| Akaza | Cancer | 2009 | Japan | 203 | 75 | Yes | T2-T4, Mx | NA | GnRH agonist + placebo CAB | 5.2 y | No | No | No | Yes |
| Bolla | NEJM | 2009 | International | 970 | 69 | Yes | T1-T4, M0 | (24%) | RT + CAB 6 mo. RT + CAB 6 mo. + GnRH agonist | 6.4 y | No | No | No | Yes |
| Calais da Silva | European Urology | 2009 | International | 626 | 73 | CPT + GnRH agonist 3 mo. | T3-T4, Mx | (10-17%) | GnRH agonist + CPT intermittent GnRH agonist + CPT continuous | 51 mo. | No | No | Yes | Yes |
| Horwitz | JCO | 2008 | USA, Canada | 1521 | 70 | Yes | T2 à T4, M0 | CVD (25-30%) Hypertension (35%) Diabetes (13-15%) | RT + CAB 4 mo. RT + CAB 4 mo. + GnRH agonist (2 years) | 11.3 y | No | No | No | Yes |
| Efstathiou | European Urology | 2008 |  |  |  |  |  |  |  | 8.1 y | No | No | Yes | No |
| Irani | European Urology | 2008 | Europe | 129 | 72 | Yes | M1 | NA | CAB intermittent CAB continuous | 42.8 mo. | No | No | No | Yes |
| Mc Roach III | JCO | 2008 | USA | 456 | 70 | Yes | B2 (30%) | NA | RT RT + short term CAB | 13.2 y | No | No | Yes | Yes |
| Iversen  (trial 24) | BJUI | 2010 | Europe, South Africa, Mexico, Australia, Israel | 3603 | 68.6 | Yes | T1-T4, Nx, M0 | NA | Placebo + standard care AA + standard care | 9.7 y | No | No | No | Yes |
| Iversen  (trial 23) | BJUI | 2010 | North America | 3292 | 64.5 | Yes | T1-T4,  M0 | NA | Placebo + standard care AA + standard care | 9.7 y | No | No | No | Yes |
| Iversen  (trial 25) | BJUI | 2010 | Scandinavia | 1218 | 68.5 | Yes | T1-T4, Nx, M0 | NA | Placebo + standard care AA + standard care | 9.7 y | No | No | No | Yes |
| Eisenberger | NEJM | 1998 | USA, Japan | 1385 | 71 | RP (12.5%) RT (4.3-6.4%) | M1 | NA | OT + placebo OT + AA | 49 mo. | No | No | No | Yes |
| Schröder^0^ | European Urology | 2004 | Europe | 310 | 70 | Yes | T0-T4, Mx | (10%) | AA CPT continuous | 8.6 y | Yes | Yes | Yes | Yes |
| Boccardo | European Urology | 2002 | Italy | 220 | 74 | Yes | T1-T4, Mx | NA | AA CAB continuous | 54 mo. | No | No | No | Yes |
| Chang | JCO | 1996 | USA | 92 | 67 | RT (25%) | D2, M1 | (36-53 %) | AA Oestrogen (DES) | 59 mo. | Yes | Yes | Yes | No |
| Aro | Ann Chir Gynaecol | 1993 | Finland | 147 | 72 | Yes | T3-T4, Mx | NA | GnRH agonist Oestrogen | 36 mo. | No | No | Yes | No |
| Denis | European Urology | 1998 | Europe | 310 | 75% > 66 | Yes | T0-T4, Mx | NA | OT CAB continuous | 7.2 y | No | No | No | Yes |
| Iversen | Cancer | 1993 | Denmark | 262 | NA | Yes | M1 | NA | OT CAB continuous | 57 mo. | No | No | Yes | Yes |
| Boccardo | Eur J Cancer | 1993 | Italy | 373 | 73 | Yes | stage C or D | NA | GnRH agonist CAB continuous | 24 mo. | No | No | No | Yes |
|  |  |  |  |  |  |  |  |  |  |  |  |  |  |  |
|  |  |  |  |  |  |  |  |  |  |  |  |  |  |  |
| First author | Journal | Publication  year | Country | Patients  N | Median age (years) | Participants naïve of treatment | T-score Metastasis | CV history | Drugs compared | Follow-up duration  (years) | Data provided on outcome | | | |
|  |  |  |  |  |  |  |  |  |  |  | MI | Stroke | CV death | Overall death |
| Kaisary | BJU | 1991 | United Kingdom | 292 | 72 | Yes | T0-T4, Mx | NA | OT GnRH agonist | 59.6 wk. | No | No | No | Yes |
| Sharifi | Urology | 1985 | USA | 25 | NA | Yes | D2, M1 | NA | GnRH agonist  Oestrogen (DES) | 72 wk. | No | No | No | Yes |
| Kotake | Japanese Journal of Clinical Oncology | 1999 | Japan | 388 | 73 | Yes | Stage C to D2 | NA | GnRH agonist GnRH agonist + CMD short term GnRH agonist + CMD long term GnRH agonist + DES short term | 3 y | No | No | No | Yes |
| Botto | Prog Clin Biol Res | 1989 | France | 80 | NA | Yes | Stage C or D | NA | OT GnRH agonist | 3 y | No | No | No | Yes |
| Lukkarinen | Scand J Urol Nephrol | 1994 | Finland | 236 | NA | Yes | T2-T4, Nx, Mx | NA | GnRH agonist Oestrogen (PEP) | 26 mo. | Yes | No | Yes | Yes |
| Organ | Am J Clin Oncol | 2013 | Canada | 31 | M1 | Yes | M1 | NA | GnRH agonist Intermittent GnRH agonist Continuous | 27.8 mo. | No | No | No | Yes |
| Crawford | NEJM | 1989 | USA | 603 | 68 | RT | M1 stage D2 | NA | GnRH agonist + placebo CAB continuous | 42 mo. | No | No | No | Yes |
| Klotz | BJUI | 2008 | International | 610 | 73 | Yes | T1-T4, Nx, M0 | NA | GnRH agonist GnRH antagonist | 12 mo. | No | No | Yes | No |
| Smith | Journal of Urology | 2010 | International | 610 | 73 | Yes | T1-T4, Nx, M0 | NA | GnRH agonist GnRH antagonist | 12 mo. | Yes | Yes | No | No |
| Akaza | BJUI | 2003 | Japan | 178 | 78 | Yes | T1-T3, M0 | NA | GnRH agonist GnRH agonist + CMD long term | 78 mo. | No | No | No | Yes |
| Manikandan | Urol Int | 2005 | United Kingdom | 58 | 76.7 | Yes | M1 (30- 50%) | NA | GnRH agonist + DES GnRH agonist + AA | 24 mo. | No | No | No | Yes |
| Crook | Int J Radiat Oncol Biol Phys | 2009 | Canada | 361 | 72 | Yes | T1-T4, M0 | NA | short term CAB long term CAB | 79 mo. | No | No | Yes | No |
| Burns-Cox | International Journal of Urology | 2002 | United Kingdom | 28 | 74 | No (OT or LHRH) | Not given | NA | AA Oestrogen (DES) | 18.3 mo. | No | No | No | Yes |
| Brisset | Prog Clin Biol Res | 1987 | France | 127 | 72 | Yes | Stage D1, D2 | NA | OT + placebo OT + AA | 18 mo. | No | No | No | Yes |
| Mikkola | BJU | 1998 | Finland | 444 | 73 | Yes | T1-T4, Mx | NA | OT Oestrogen (PEP) | 2 y | Yes | Yes | Yes | No |
| Waymont | BJU | 1992 | United Kingdom | 250 | 72.5 | Yes | T3-T4, Mx | NA | GnRH agonist  Oestrogen (DES) | 43 mo. | Yes | Yes | No | Yes |
| Thorpe | European Urology | 1996 | United Kingdom | 525 | 71 | Yes | T0-T4, Mx | (26-33%) | CPT long term GnRH agonist CPT long term + GnRH agonist | 4 y | No | No | No | Yes |
| Robinson | European Urology | 1995 | Europe | 351 | 85% > 65 | Yes | T0-T4, Mx | Stroke (1-2%) IHD (5-10%) MI (3-7%) | OT OT + CPT DES | 4 y | Yes | Yes | Yes | Yes |
| Armstrong | Int J Radiat Oncol Biol Phys | 2011 | Ireland | 261 | 67 | OT | T1-T4, M0 | NA | RT RT + short term CAB | 102 mo. | No | No | No | Yes |
| Dijkman | The Journal of Urology | 1997 | Netherlands | 457 | NA | OT | Stage D2 | NA | OT + placebo OT + AA | 8.5 y | No | No | No | Yes |
| Ostri | Urol Int | 1991 | Denmark | 37 | 74 | Yes | T1-T4, Nx, Mx | NA | OT CPT | 12 mo. | No | No | No | Yes |
| Wirth | European Urologia | 2004 | Germany | 309 | 64 | No | T3-T4, M0 | NA | No treatment AA | 6.1 y | No | No | No | Yes |
| Bales | Urology | 1996 | Scandinavian | 376 | 71 | Yes | stage D2 | NA | OT AA | 17 mo. | No | No | No | Yes |
| Citrin | The Prostate | 1991 | USA | 77 | 69 | Yes | Stage D2 | NA | GnRH agonist Oestrogen (DES) | 95 wk. | No | No | No | Yes |
| Ansari | Int J Urol | 2004 | India | 100 | 60 | Yes | stage D2 | NA | OT OT + AA | 3.5 y | No | No | No | Yes |
| Pavone-Macaluso | The Journal of Urology | 1986 | Europe | 210 | 90% > 60 | Yes | T1-T4, Mx | 27% | CPT Oestrogen (DES) Medroxyprogesterone | 7 y | No | No | No | Yes |
| Parmar | Am J Clin Oncol | 1988 | United Kingdom | 110 | NA | Yes | M1 | NA | OT GnRH agonist | 45 mo. | No | No | No | Yes |
|  |  |  |  |  |  |  |  |  |  |  |  | | | |
|  |  |  |  |  |  |  |  |  |  |  |  | | | |
| First author | Journal | Publication  year | Country | Patients  N | Median age (years) | Participants naïve of treatment | T-score Metastasis | CV history | Drugs compared | Follow-up duration  (years) | Data provided on outcome | | | |
|  |  |  |  |  |  |  |  |  |  |  | MI | Stroke | CV death | Overall death |
| Tyrrell | European Urologia | 2000 | Europe | 586 | 73 | Yes | T3-T4, Mx | NA | GnRH agonist CAB continuous | 4.9 y | No | No | No | Yes |
| Bono | Urol Int | 1998 | Italy | 241 | 68 | Yes | Stage C-D1, (M1 75%) | (25 à 30%) | GnRH agonist CAB continuous | 44 mo. | No | No | Yes | No |
| Zalcberg | Br J Urol | 1996 | Australia | 222 | 72 | RT (24-34%) | Stage D | (53%) | OT + Placebo OT + AA | 60 mo. | No | No | Yes | No |
| Navratil | Prog Clin Biol Res | 1987 | France | 38 | 72 | Yes | Stage D2 | NA | GnRH agonist CAB continuous | 24 mo. | No | No | No | Yes |
| Anderson | Urol Int | 2013 | Europe | 40 | 70 | 5ARI, adreno-receptor antagonist | T1-T4, Mx | NA | GnRH antagonist Short term CAB | 12 wk. | No | No | No | Yes |

AA: antiandrogen – BT: brachytherapy – CAB: Combined androgen blockade = GnRH agonist + antiandrogen – CPT: cyproterone – DES: diethylstilbestrol – OT: orchidectomy – PEP: polyestradiol phosphate – RT: radiotherapy.

NA: not available.

**Bibliography:**

- Akaza H, Homma Y, Okada K, et al. A prospective and randomized study of primary hormonal therapy for patients with localized or locally advanced prostate cancer unsuitable for radical prostatectomy: results of the 5-year follow-up. BJU Int. 2003;91(1):33-36.

- Akaza H, Hinotsu S, Usami M, et al. Combined androgen blockade with bicalutamide for advanced prostate cancer: Long-term follow-up of a phase 3, double-blind, randomized study for survival. Cancer. 2009;115(15):3437-3445.

- Anderson J, Al-Ali G, Wirth M, et al. Degarelix versus goserelin (+ antiandrogen flare protection) in the relief of lower urinary tract symptoms secondary to prostate cancer: results from a phase IIIb study (NCT00831233). Urol Int. 2013;90(3):321-328.

- Ansari MS, Gupta NP, Hemal AK, Dogra PN, Seth A. Combined androgen blockade in the management of advanced prostate cancer: a sensible or ostensible approach. Int J Urol Off J Jpn Urol Assoc. 2004;11(12):1092-1096.

- Armstrong JG, Gillham CM, Dunne MT, et al. A randomized trial (Irish clinical oncology research group 97-01) comparing short versus protracted neoadjuvant hormonal therapy before radiotherapy for localized prostate cancer. Int J Radiat Oncol Biol Phys. 2011;81(1):35-45.

- Aro J, Ruutu M, Juusela H, Hansson E, Permi J. Polyestradiol phosphate (160 mg/month) or LHRH analog (buserelin depot) in the treatment of locally advanced or metastasized prostatic cancer. The Finnprostate Group. Ann Chir Gynaecol Suppl. 1993;206:5-8.

- Bales GT, Chodak GW. A controlled trial of bicalutamide versus castration in patients with advanced prostate cancer. Urology. 1996;47(1A Suppl):38-43; discussion 48-53.

- Boccardo F, Pace M, Rubagotti A, et al. Goserelin acetate with or without flutamide in the treatment of patients with locally advanced or metastatic prostate cancer. Eur J Cancer. 1993;29(8):1088-1093.

- Boccardo F, Barichello M, Battaglia M, et al. Bicalutamide monotherapy versus flutamide plus goserelin in prostate cancer: updated results of a multicentric trial. Eur Urol. 2002;42(5):481-490.

- Bolla M, de Reijke TM, Van Tienhoven G, et al. Duration of androgen suppression in the treatment of prostate cancer. N Engl J Med. 2009;360(24):2516-2527..

- Bolla M, Van Tienhoven G, Warde P, et al. External irradiation with or without long-term androgen suppression for prostate cancer with high metastatic risk: 10-year results of an EORTC randomised study. Lancet Oncol. 2010;11(11):1066-1073.

- Bono AV, DiSilverio F, Robustelli della Cuna G, et al. Complete androgen blockade versus chemical castration in advanced prostatic cancer: analysis of an Italian multicentre study. Italian Leuprorelin Group. Urol Int. 1998;60 Suppl 1:18-24.

- Botto H, Richard F, Mathieu F, Camey M. Decapeptyl in the treatment of advanced prostatic cancer: comparative study with pulpectomy. Prog Clin Biol Res. 1989;303:53-60.

- Brisset JM, Boccon-Gibod L, Botto H, et al. Anandron (RU 23908) associated to surgical castration in previously untreated stage D prostate cancer: a multicenter comparative study of two doses of the drug and of a placebo. Prog Clin Biol Res. 1987;243 A:411-422

- Burns-Cox N, Basketter V, Higgins B, Holmes S. Prospective randomised trial comparing diethylstilboestrol and flutamide in the treatment of hormone relapsed prostate cancer. Int J Urol Off J Jpn Urol Assoc. 2002;9(8):431-434.

- Bruun E, Frimodt-Møller C. The effect of Buserelin versus conventional antiandrogenic treatment in patients with T2-4NXM1 prostatic cancer. A prospective, randomized multicentre phase III trial. The “Danish Buserelin Study Group.” Scand J Urol Nephrol. 1996;30(4):291-297.

- Calais da Silva FEC, Bono AV, Whelan P, et al. Intermittent androgen deprivation for locally advanced and metastatic prostate cancer: results from a randomised phase 3 study of the South European Uroncological Group. Eur Urol. 2009;55(6):1269-1277.

- Chang A, Yeap B, Davis T, et al. Double-blind, randomized study of primary hormonal treatment of stage D2 prostate carcinoma: flutamide versus diethylstilbestrol. J Clin Oncol Off J Am Soc Clin Oncol. 1996;14(8):2250-2257.

- Citrin DL, Resnick MI, Guinan P, et al. A comparison of Zoladex and DES in the treatment of advanced prostate cancer: results of a randomized, multicenter trial. The Prostate. 1991;18(2):139-146.

- Crawford ED, Eisenberger MA, McLeod DG, et al. A controlled trial of leuprolide with and without flutamide in prostatic carcinoma. N Engl J Med. 1989;321(7):419-424.

- Crook J, Ludgate C, Malone S, et al. Final report of multicenter Canadian Phase III randomized trial of 3 versus 8 months of neoadjuvant androgen deprivation therapy before conventional-dose radiotherapy for clinically localized prostate cancer. Int J Radiat Oncol Biol Phys. 2009;73(2):327-333.

- D’Amico AV, Chen M-H, Renshaw AA, Loffredo M, Kantoff PW. Androgen suppression and radiation vs radiation alone for prostate cancer: a randomized trial. JAMA. 2008;299(3):289-295.

- Denham JW, Steigler A, Lamb DS, et al. Short-term neoadjuvant androgen deprivation and radiotherapy for locally advanced prostate cancer: 10-year data from the TROG 96.01 randomised trial. Lancet Oncol. 2011;12(5):451-459.

- Denis LJ, Keuppens F, Smith PH, et al. Maximal androgen blockade: Final analysis of EORTC phase III trial 30853. Eur Urol. 1998;33(2):144-151.

- De Voogt HJ, Studer U, Schroder FH, Klijn JG, De Pauw M, Sylvester R. Maximum androgen blockade using LHRH agonist buserelin in combination with short-term (two weeks) or long-term (continuous) cyproterone acetate is not superior to standard androgen deprivation in the treatment of advanced prostate cancer. Final analysis of EORTC GU group trial 30843. Eur Urol. 1998;33(2):152-158.

- Dijkman GA, Janknegt RA, De Reijke TM, Debruyne FM. Long-term efficacy and safety of nilutamide plus castration in advanced prostate cancer, and the significance of early prostate specific antigen normalization. International Anandron Study Group. J Urol. 1997;158(1):160-163.

- Efstathiou JA, Bae K, Shipley WU, et al. Cardiovascular mortality and duration of androgen deprivation for locally advanced prostate cancer: analysis of RTOG 92-02. Eur Urol. 2008;54(4):816-823.

- Eisenberger MA, Blumenstein BA, Crawford ED, et al. Bilateral orchiectomy with or without flutamide for metastatic prostate cancer. N Engl J Med. 1998;339(15):1036-1042.

- Horwitz EM, Bae K, Hanks GE, et al. Ten-year follow-up of radiation therapy oncology group protocol 92-02: A phase III trial of the duration of elective androgen deprivation in locally advanced prostate cancer. J Clin Oncol. 2008;26(15):2497-2504.

- Hussain M, Tangen CM, Berry DL, et al. Intermittent versus continuous androgen deprivation in prostate cancer. N Engl J Med. 2013;368(14):1314-1325.

- Irani J, Celhay O, Hubert J, et al. Continuous versus six months a year maximal androgen blockade in the management of prostate cancer: a randomised study. Eur Urol. 2008;54(2):382-391.

- Iversen P, Rasmussen F, Klarskov P, Christensen IJ. Long-term results of Danish Prostatic Cancer Group trial 86. Goserelin acetate plus flutamide versus orchiectomy in advanced prostate cancer. *Cancer* 1993; **72**: 3851–4.

- Iversen P, McLeod DG, See WA, et al. Antiandrogen monotherapy in patients with localized or locally advanced prostate cancer: final results from the bicalutamide Early Prostate Cancer programme at a median follow-up of 9.7 years. BJU Int. 2010;105(8):1074-1081.

- Jones CU, Hunt D, McGowan DG, et al. Radiotherapy and short-term androgen deprivation for localized prostate cancer. N Engl J Med. 2011;365(2):107-118.

- Kaisary AV, Tyrrell CJ, Peeling WB, Griffiths K. Comparison of LHRH analogue (Zoladex) with orchiectomy in patients with metastatic prostatic carcinoma. Br J Urol. 1991;67(5):502-508.

- Klotz L, Boccon-Gibod L, Shore ND, et al. The efficacy and safety of degarelix: A 12-month, comparative, randomized, open-label, parallel-group phase III study in patients with prostate cancer. BJU Int. 2008;102(11):1531-1538.

- Kotake T, Usami M, Akaza H, et al. Goserelin acetate with or without antiandrogen or estrogen in the treatment of patients with advanced prostate cancer: A multicenter, randomized, controlled trial in Japan. Jpn J Clin Oncol. 1999;29(11):562-570.

- Lukkarinen O, Kontturi M. Comparison of a long-acting LHRH agonist and polyoestradiol phosphate in the treatment of advanced prostatic carcinoma. An open prospective, randomized multicentre study. Scand J Urol Nephrol. 1994;28(2):171-178.

- Manikandan R, Srirangam SJ, Pearson E, Brown SCW, O’Reilly P, Collins GN. Diethylstilboestrol versus bicalutamide in hormone refractory prostate carcinoma: a prospective randomized trial. Urol Int. 2005;75(3):217-221.

- Mikkola AK, Ruutu ML, Aro JL, Rannikko SA, Salo JO. Parenteral polyoestradiol phosphate vs orchidectomy in the treatment of advanced prostatic cancer. Efficacy and cardiovascular complications: a 2-year follow-up report of a national, prospective prostatic cancer study. Finnprostate Group. Br J Urol. 1998;82(1):63-68.

- Mottet N, Van Damme J, Loulidi S, et al. Intermittent hormonal therapy in the treatment of metastatic prostate cancer: a randomized trial. BJU Int. 2012;110(9):1262-1269.

- Navratil H. Double-blind study of Anandron versus placebo in stage D2 prostate cancer patients receiving buserelin. Results on 49 cases from a multicentre study. Prog Clin Biol Res. 1987;243A:401-410.

- Organ M, Wood L, Wilke D, et al. Intermittent LHRH therapy in the management of castrate-resistant prostate cancer (CRPCa): results of a multi-institutional randomized prospective clinical trial. Am J Clin Oncol. 2013;36(6):601-605.

- Ostri P, Bonnesen T, Nilsson T, Frimodt-Møller C. Treatment of symptomatic metastatic prostatic cancer with cyproterone acetate versus orchiectomy: a prospective randomized trial. Urol Int. 1991;46(2):167-171.

- Parmar H, Phillips RH, Lightman SL, Edwards L. How would you like to have an orchidectomy for advanced prostatic cancer? Am J Clin Oncol. 1988;11 Suppl 2:S160-S168.

- Pavone-Macaluso M, de Voogt HJ, Viggiano G, et al. Comparison of diethylstilbestrol, cyproterone acetate and medroxyprogesterone acetate in the treatment of advanced prostatic cancer: Final analysis of a randomized phase III trial of the European Organization for Research on Treatment of Cancer Urological Group. J Urol. 1986;136(3):624-631.

- Roach III M, Bae K, Speight J, et al. Short-term neoadjuvant androgen deprivation therapy and external-beam radiotherapy for locally advanced prostate cancer: Long-term results of RTOG 8610. J Clin Oncol. 2008;26(4):585-591.

- Robinson MR, Smith PH, Richards B, Newling DW, de Pauw M, Sylvester R. The final analysis of the EORTC Genito-Urinary Tract Cancer Co-Operative Group phase III clinical trial (protocol 30805) comparing orchidectomy, orchidectomy plus cyproterone acetate and low dose stilboestrol in the management of metastatic carcinoma of the prostate. Eur Urol. 1995;28(4):273-283.

- Sharifi R, Lee M, Ojeda L, Ray P, Stobnicki M, Guinan P. Comparison of leuprolide and diethylstilbestrol for stage D2 adenocarcinoma of prostate. Urology. 1985;26(2):117-124.

- Schröder FH, Whelan P, De Reijke TM, et al. Metastatic prostate cancer treated by Flutamide versus Cyproterone acetate: Final analysis of the “European Organization for Research and Treatment of Cancer” (EORTC) protocol 30892. Eur Urol. 2004;45(4):457-464.

- Smith MR, Klotz L, Persson B-E, Olesen TK, Wilde AAM. Cardiovascular safety of degarelix: results from a 12-month, comparative, randomized, open label, parallel group phase III trial in patients with prostate cancer. J Urol. 2010;184(6):2313-2319.

- Thorpe SC, Azmatullah S, Fellows GJ, Gingell JC, O’Boyle PJ. A prospective, randomised study to compare goserelin acetate (Zoladex(®)) versus cyproterone acetate (Cyprostat(®)) versus a combination of the two in the treatment of metastatic prostatic carcinoma. Eur Urol. 1996;29(1):47-54.

- Tyrrell CJ, Altwein JE, Klippel F, et al. Comparison of an LH-RH analogue (Goeserelin acetate, “Zoladex”) with combined androgen blockade in advanced prostate cancer: final survival results of an international multicentre randomized-trial. International Prostate Cancer Study Group. Eur Urol. 2000;37(2):205-211.

- Vogelzang NJ, Chodak GW, Soloway MS, et al. Goserelin versus orchiectomy in the treatment of advanced prostate cancer: final results of a randomized trial. Zoladex Prostate Study Group. Urology. 1995;46(2):220-226.

- Waymont B, Lynch TH, Dunn JA, et al. Phase III randomised study of zoladex versus stilboestrol in the treatment of advanced prostate cancer. Br J Urol. 1992;69(6):614-620

- Wirth MP, Weissbach L, Marx F-J, et al. Prospective randomized trial comparing flutamide as adjuvant treatment versus observation after radical prostatectomy for locally advanced, lymph node-negative prostate cancer. Eur Urol. 2004;45(3):267-270; discussion 270.

- Zalcberg JR, Raghaven D, Marshall V, Thompson PJ. Bilateral orchidectomy and flutamide versus orchidectomy alone in newly diagnosed patients with metastatic carcinoma of the prostate--an Australian multicentre trial. Br J Urol. 1996;77(6):865-869.

**Appendix Table 3. Overall results from observational studies with direct meta-analyses.**

| Outcome | Reference | Tested therapy | Comparisons, n | Relative Risk | 95% LCL | 95% UCL | I² |
| --- | --- | --- | --- | --- | --- | --- | --- |
| Myocardial infarction | AA | OT | 1 | 2.04 | 0.66 | 8.33 |  |
|  | OT | GnRH agonist | 1 | 0.61 | 0.30 | 0.92 |  |
|  | OT | CAB | 1 | 0.49 | 0.19 | 0.89 |  |
|  | GnRH agonist | CAB | 4 | 0.97 | 0.63 | 1.47 | 86% |
|  | AA | GnRH agonist | 4 | 1.43 | 1.10 | 1.85 | 59% |
|  | AA | CAB | 4 | 1.34 | 0.87 | 2.06 | 78% |
|  | No endocrine treatment | GnRH agonist | 4 | 1.41 | 1.19 | 1.68 | 77% |
|  | AA | No endocrine treatment | 4 | 0.91 | 0.79 | 1.05 | 0% |
|  | No endocrine treatment | CAB | 4 | 1.27 | 0.90 | 1.79 | 78% |
|  | No endocrine treatment | OT | 2 | 2.11 | 1.30 | 3.42 | 0% |
| Stroke | AA | OT | 3 | 1.14 | 0.83 | 1.56 | 0% |
|  | OT | GnRH agonist | 3 | 1.00 | 0.58 | 1.72 | 84% |
|  | OT | CAB | 3 | 0.71 | 0.52 | 0.97 | 0% |
|  | GnRH agonist | CAB | 4 | 0.82 | 0.66 | 1.02 | 70% |
|  | AA | GnRH agonist | 4 | 1.22 | 0.93 | 1.61 | 77% |
|  | AA | CAB | 4 | 1.10 | 1.02 | 1.19 | 4% |
|  | No endocrine treatment | OT | 3 | 1.68 | 1.27 | 2.22 | 0% |
|  | No endocrine treatment | GnRH agonist | 3 | 1.22 | 1.11 | 1.34 | 0% |
|  | AA | No endocrine treatment | 3 | 0.99 | 0.66 | 1.49 | 75% |
|  | No endocrine treatment | CAB | 3 | 0.88 | 0.59 | 1.32 | 78% |
| CV death | No endocrine treatment | CAB ≤ 6 months | 1 | 1.01*^^ | 0.74 | 1.39 |  |
|  | No endocrine treatment | CAB > 6 months | 1 | 1.04*^^ | 0.65 | 1.67 |  |
| All-cause mortality | No endocrine treatment | CAB 6 months | 1 | 0.30^^ | 0.16 | 0.58 |  |
|  | No endocrine treatment | CAB ≤ 6 months | 1 | 1.12*^^ | 0.77 | 1.62 |  |
|  | No endocrine treatment | CAB > 6 months | 1 | 1.03*^^ | 0.75 | 1.41 |  |
|  | No endocrine treatment | CAB (1 to 96 months) | 1 | 1.02 | 0.98 | 1.05 |  |
|  | CAB | GnRH agonist | 1 | 1.26^^ | 0.78 | 2.03 |  |
|  | OT + AA | CAB | 1 | 0.57^^ | 0.07 | 4.38 |  |
|  | OT | GnRH agonist | 1 | 1.12 | 0.64 | 1.96 |  |
|  | No endocrine treatment** | GnRH agonist | 1 | ◊ |  |  |  |

AA: antiandrogen – CAB: Combined androgen blockade = GnRH agonist + antiandrogen – OT: orchidectomy.

* Authors stratified the data on the risk of death in the prostate cancer population. Low and intermediate risk represented the most of the population and the RR chosen was this one.

** No endocrine treatment: All patients underwent brachytherapy >3 years before analysis and some received supplemental radiation therapy.

OT means orchiectomy, AA anti-androgens, GnRH gonadotrophin releasing hormone, CAB combined androgen blockade; LCL denotes (lower limit) and UCL (upper limit)

◊ Stratification following comorbidities has be done in a study ^66^. Adjusted HR of death in group ‘MI or CHF, with revascularization’: 2.06 [1.02-4.17]; adjusted HR of death in group with no comorbidity: 0.97 [0.82-1.15].

^^ We recalculated crude relative risk from raw data, except those tagged by a “^^”.

**Appendix eTable 4. Johanna Briggs quality assessment**

☹ = 1, not specified or not realized

😐 = 2, unclear

☺ = 3, done

**Observational studies**

| Author | Year | Analysis | Is the sample representative of patients in the population as a whole? | Are the patients at a similar point in the course of their condition/illness? | Are confounding factors identified and strategies to deal with them stated? | Was follow up carried out over a sufficient time period ? | Are outcomes assessed using objective criteria? | Were the outcomes of people who withdrew described and included in the analysis? | Were outcomes measured in a reliable way? | Was appropriate statistical analysis used? | Score final |
| --- | --- | --- | --- | --- | --- | --- | --- | --- | --- | --- | --- |
| D’Amico | 2006 | Main | 😐 | ☺ | ☺ | ☺ | ☺ | ☺ | ☺ | ☺ | 23 |
| Robinson | 2012 | Main | ☺ | ☹ | ☺ | 😐 | 😐 | ☺ | ☺ | ☺ | 20 |
| Bittner | 2008 | Main | ☹ | ☹ | ☺ | ☺ | 😐 | ☹ | 😐 | ☺ | 21 |
| Nanda | 2012 | Main | ☹ | ☹ | ☺ | ☺ | ☺ | ☺ | ☺ | ☺ | 20 |
| Matsumoto | 2014 | Main | ☺ | ☹ | ☺ | ☺ | ☺ | ☺ | ☺ | ☺ | 22 |
| Van Hemelrijck | 2010 | Main | ☺ | ☹ | ☺ | ☺ | 😐 | ☹ | 😐 | ☺ | 18 |
| Koutsilieris | 1986 | Main | ☹ | ☺ | ☺ | ☺ | ☺ | ☺ | ☺ | ☺ | 22 |
| Parekh | 2013 | Main | ☺ | ☹ | ☺ | ☺ | ☺ | ☺ | ☺ | ☺ | 22 |
| Keating | 2010 | Main | ☺ | ☺ | ☺ | 😐 | 😐 | ☹ | 😐 | ☺ | 19 |
| Azoulay | 2011 | Main | ☺ | ☺ | ☺ | ☺ | 😐 | ☹ | 😐 | ☺ | 20 |
| Martin-Merino | 2011 | Main | ☺ | ☺ | ☺ | ☺ | 😐 | ☹ | 😐 | ☺ | 20 |

Using the Johanna Briggs manual^1^.

1 The Joanna Briggs Institute, The University of Adelaide. Joanna Briggs Institute. Reviewer’s manual - 2011 Edition. 2011 http://joannabriggs.org/assets/docs/sumari/ReviewersManual-2011.pdf.

**Bibliography**

- Azoulay L, Yin H, Benayoun S, Renoux C, Boivin J-F, Suissa S. Androgen-deprivation therapy and the risk of stroke in patients with prostate cancer. Eur Urol. 2011;60(6):1244-1250.

- Bittner N, Merrick GS, Galbreath RW, et al. Primary Causes of Death After Permanent Prostate Brachytherapy. Int J Radiat Oncol Biol Phys. 2008;72(2):433-440.

- D’Amico AV, Loffredo M, Renshaw AA, Loffredo B, Chen M-H. Six-month androgen suppression plus radiation therapy compared with radiation therapy alone for men with prostate cancer and a rapidly increasing pretreatment prostate-specific antigen level. J Clin Oncol. 2006;24(25):4190-4195.

- Keating NL, O’Malley AJ, Freedland SJ, Smith MR. Diabetes and cardiovascular disease during androgen deprivation therapy: Observational study of veterans with prostate cancer. J Natl Cancer Inst. 2010;102(1):39-46.

- Koutsilieris M, Faure N, Tolis G, Laroche B, Robert G, Ackman CF. Objective response and disease outcome in 59 patients with stage D2 prostatic cancer treated with either Buserelin or orchiectomy. Disease aggressivity and its association with response and outcome. Urology. 1986;27(3):221-228.

- Martín-Merino E, Johansson S, Morris T, García Rodríguez LA. Androgen deprivation therapy and the risk of coronary heart disease and heart failure in patients with prostate cancer: A nested case-control study in UK primary care. Drug Saf. 2011;34(11):1061-1077.

- Matsumoto K, Hagiwara M, Tanaka N, et al. Survival following primary androgen deprivation therapy for localized intermediate- or high-risk prostate cancer: comparison with the life expectancy of the age-matched normal population. Med Oncol Northwood Lond Engl. 2014;31(6):979.

- Nanda A, Chen M-H, Moran BJ, Braccioforte MH, D’Amico AV. Cardiovascular comorbidity and mortality in men with prostate cancer treated with brachytherapy-based radiation with or without hormonal therapy. Int J Radiat Oncol Biol Phys. 2013;85(5):e209-e215.

- Parekh A, Chen M-H, D’Amico AV, et al. Identification of comorbidities that place men at highest risk of death from androgen deprivation therapy before brachytherapy for prostate cancer. Brachytherapy. 2013;12(5):415-421..

- Robinson D, Garmo H, Lindahl B, et al. Ischemic heart disease and stroke before and during endocrine treatment for prostate cancer in PCBaSe Sweden. Int J Cancer. 2012;130(2):478-487.

- Van Hemelrijck M, Garmo H, Holmberg L, et al. Absolute and relative risk of cardiovascular disease in men with prostate cancer: Results from the population-based PCBaSe Sweden. J Clin Oncol. 2010;28(21):3448-3456.

**Appendix Table 5. Overall results from randomized controlled trials with direct meta-analyses.**

| Outcome | Reference | Tested therapy | Comparisons, n | Relative Risk | 95% LCL | 95% UCL | I² |
| --- | --- | --- | --- | --- | --- | --- | --- |
| Myocardial infarction | AA | CPT | 1 | 0.49 | 0.04 | 5.39 |  |
|  | AA | Estrogen | 1 | ∞ |  |  |  |
|  | Estrogen | GnRH agonist | 2 | 0.33 | 0.04 | 2.52 | NA* |
|  | GnRH agonist | GnRH antagonist | 1 | 0.42 | 0.23 | 0.77 |  |
|  | OT | Estrogen | 2 | 1.44 | 0.61 | 3.42 | 0% |
|  | OT | OT + CPT | 1 | 1.24 | 0.34 | 4.48 |  |
|  | OT + CPT | Estrogen | 1 | 1.04 | 0.31 | 3.48 |  |
| Stroke | AA | CPT | 1 | 0.79 | 0.22 | 2.89 |  |
|  | AA | estrogen | 1 | 0.36 | 0.04 | 3.37 |  |
|  | GnRH agonist | GnRH antagonist | 1 | 3.44 | 0.43 | 27.8 |  |
|  | estrogen | GnRH agonist | 1 | ∞ |  |  |  |
|  | OT | estrogen | 2 | 2.62 | 0.37 | 18.4 | NA* |
|  | OT | OT + CPT | 1 | 0.49 | 0.05 | 5.37 |  |
|  | OT + CPT | estrogen | 1 | 3.12 | 0.33 | 29.5 |  |
| CV death | CAB short term | CAB long term | 2 | 1.16 | 0.55 | 2.41 | 0% |
|  | GnRH agonist | CAB | 1 | 1.00 | 0.85 | 1.19 |  |
|  | Estrogen | GnRH agonist | 2 | 0.94 | 0.42 | 2.07 | 0% |
|  | CPT intermittent | CPT continuous | 1 | 1.23^^ | 0.81 | 1.87 |  |
|  | AA | CPT continuous | 1 | 0.77 | 0.40 | 1.49 |  |
|  | AA | Estrogen | 1 | 1.85 | 0.17 | 19.6 |  |
|  | OT | CAB continuous | 1 | 1.44 | 0.47 | 4.43 |  |
|  | OT | GnRH agonist | 2 | 1.11 | 0.85 | 1.44 | 0% |
|  | OT | Estrogen** | 2 | 1.69 | 0.55 | 5.21 | 0% |
|  | OT | OT + CPT | 1 | 1.29 | 0.56 | 2.93 |  |
|  | OT | GnRH agonist + CPT long term | 1 | 0.80 | 0.35 | 1.82 |  |
|  | OT | OT + AA | 1 | 1.02 | 0.86 | 1.20 |  |
|  | OT + CPT | estrogen | 1 | 1.38 | 0.69 | 2.78 |  |
| All-cause mortality | intermittent CAB | Continuous CAB | 3 | 0.91 | 0.81 | 1.02 | 0% |
|  | CAB short term | CAB long term | 2 | 0.88 | 0.74 | 1.05 | 80% |
|  | CPT intermittent | CPT continuous | 1 | 0.99^^ | 0.80 | 1.23 |  |
|  | OT | OT + AA | 4 | 0.90 | 0.83 | 0.97 | 0% |
|  | AA | CPT continuous | 1 | 1.22^^ | 0.95 | 1.57 |  |
|  | AA | CAB continuous | 1 | 0.93^^ | 0.64 | 1.35 |  |
|  | OT | GnRH agonist | 5 | 0.93 | 0.86 | 1.00 | 0% |
|  | OT | Estrogen | 2 | 0.95 | 0.83 | 1.09 | 77% |
|  | Estrogen | GnRH agonist | 5 | 1.05 | 0.88 | 1.24 | 20% |
|  | OT | CAB continuous | 2 | 0.94 | 0.85 | 1.05 | 68% |
|  | GnRH agonist | CAB continuous | 5 | 0.90 | 0.82 | 1.00 | 60% |
|  | OT | GnRH agonist + CPT long term | 1 | 0.96 | 0.87 | 1.06 |  |
|  | Estrogen long term | CMD short term | 1 | 1.18 | 0.80 | 1.75 |  |
|  | Estrogen long term | CMD long term | 1 | 1.48 | 1.03 | 2.12 |  |
|  | CMD short term | CMD long term | 1 | 1.25 | 0.89 | 1.75 |  |
|  | AA | OT | 1 | 0.57^^ | 0.41 | 0.79 |  |
|  | GnRH agonist intermittent | GnRH agonist concomitant | 1 | 1.04 | 0.83 | 1.30 |  |

Appendix Table 5 (continued).

| Outcome | Reference | Tested therapy | Comparisons, n | Relative Risk | 95% LCL | 95% UCL | I² |
| --- | --- | --- | --- | --- | --- | --- | --- |
|  | GnRH agonist | GnRH antagonist | 1 | 0.55 | 0.22 | 1.32 |  |
|  | AA | Estrogen | 2 | 0.90 | 0.70 | 1.16 | 0% |
|  | GnRH agonist | CPT continuous | 1 | 1.39 | 0.79 | 2.45 |  |
|  | OT | OT + CPT | 1 | 1.00 | 0.88 | 1.14 |  |
| All-cause mortality | OT + CPT | Estrogen | 1 | 0.97 | 0.85 | 1.11 |  |
|  | OT | CPT | 1 | 1.49 | 0.74 | 2.98 |  |
|  | Estrogen | CPT | 1 | 1.09 | 0.79 | 1.51 |  |
|  | Estrogen | MPA | 1 | 1.35 | 1.01 | 1.81 |  |
|  | CPT | MPA | 1 | 0.81 | 0.62 | 1.05 |  |
|  | CAB short term | GnRH antagonist | 1 | ∞ |  |  |  |

*This estimation was done using one study which included arm without event.

AA denotes antiandrogens; CPT, cyproterone acetate; OT, orchiectomy; CAB, combined androgen blockade (agonist LHRH + antiandrogen) with short term defined as 3 months of treatment, but long term had different definition across studies: 6 months in one study and 8 months in the second one.

DES denotes Diethylstilbestrol; MPA, Medroxy Progesterone acetate,

** DES in one study and PEP in the other.

^^ We recalculated crude relative risk from raw data, except those tagged with “^^”.

**Appendix eTable 6. Johanna Brigg’s quality assessment.**

☹ = 1, unclear or not specified or not realized

☺ = 3, done

**Randomized Controlled Trials**

| Author | Year | Analysis | Was the assignment to treatment groups truly random? | Were participants blinded to treatment allocation? | Was allocation to treatment groups concealed from the allocator? | Were the outcomes of people who withdrew described and included in the analysis? | Were those assessing outcomes blind to the treatment allocation? | Were the control and treatment groups comparable at entry? | Were groups treated identically other than for the named interventions?* | Were outcomes measured in the same way for all groups? | Were outcomes measured in a reliable way? | Was appropriate statistical analysis used? | Score final |
| --- | --- | --- | --- | --- | --- | --- | --- | --- | --- | --- | --- | --- | --- |
| D’Amico | 2008 | Main | ☺ | ☹ | ☺ | ☺ | ☺ | ☺ | ☺ | ☺ | ☺ | ☺ | 28 |
| Hussain | 2013 | Main | ☺ | ☹ | ☹ | ☹ | ☺ | ☺ | ☹ | ☺ | ☺ | ☺ | 22 |
| Mottet | 2011 | Main | ☺ | ☹ | ☹ | ☺ | ☺ | ☺ | ☺ | ☺ | ☺ | ☺ | 26 |
| Jones | 2011 | Main | ☺ | ☹ | ☹ | ☹ | ☹ | ☺ | ☹ | ☺ | ☺ | ☺ | 20 |
| Denham | 2011 | Main | ☺ | ☹ | ☹ | ☹ | ☹ | ☺ | ☹ | ☺ | ☺ | ☺ | 20 |
| Bolla | 2010 | Main | ☺ | ☹ | ☺ | ☹ | ☺ | ☺ | ☹ | ☺ | ☺ | ☺ | 24 |
| Akaza | 2009 | Main | ☺ | ☺ | ☺ | ☹ | ☺ | ☺ | ☹ | ☺ | ☺ | ☺ | 28 |
| Bolla | 2009 | Main | ☺ | ☹ | ☺ | ☹ | ☺ | ☺ | ☹ | ☺ | ☺ | ☺ | 22 |
| Calais da Silva | 2009 | Main | ☺ | ☹ | ☹ | ☹ | ☹ | ☺ | ☺ | ☺ | ☺ | ☺ | 22 |
| Horwitz | 2008 | Main | ☺ | ☹ | ☺ | ☹ | ☺ | ☺ | ☺ | ☺ | ☺ | ☺ | 28 |
| Irani | 2008 | Main | ☺ | ☹ | ☹ | ☹ | ☹ | ☺ | ☺ | ☺ | ☺ | ☺ | 22 |
| Roach | 2008 | Main | ☺ | ☹ | ☹ | ☹ | ☹ | ☺ | ☺ | ☺ | ☺ | ☺ | 22 |
| Iversen  (Trial 23) | 2010 | Main | ☺ | ☺ | ☹ | ☺ | ☹ | ☺ | ☹ | ☺ | ☺ | ☺ | 24 |
| Iversen  (Trial 24) | 2010 | Main | ☺ | ☺ | ☹ | ☺ | ☹ | ☺ | ☹ | ☺ | ☺ | ☺ | 24 |
| Iversen  (Trial 25) | 2010 | Main | ☺ | ☺ | ☹ | ☺ | ☹ | ☺ | ☹ | ☺ | ☺ | ☺ | 24 |
| Eisenberger | 1998 | Main | ☺ | ☺ | ☺ | ☹ | ☺ | ☺ | ☺ | ☺ | ☺ | ☺ | 28 |
|  |  |  |  |  |  |  |  |  |  |  |  |  |  |
|  |  |  |  |  |  |  |  |  |  |  |  |  |  |
| Author | Year | Analysis | Was the assignment to treatment groups truly random? | Were participants blinded to treatment allocation? | Was allocation to treatment groups concealed from the allocator? | Were the outcomes of people who withdrew described and included in the analysis? | Were those assessing outcomes blind to the treatment allocation? | Were the control and treatment groups comparable at entry? | Were groups treated identically other than for the named interventions? | Were outcomes measured in the same way for all groups? | Were outcomes measured in a reliable way? | Was appropriate statistical analysis used? | Score final |
| Schröder | 2004 | Main | ☺ | ☹ | ☹ | ☺ | ☹ | ☺ | ☹ | ☺ | ☺ | ☺ | 25 |
| Boccardo | 2002 | Main | ☺ | ☹ | ☹ | 😐 | ☹ | ☺ | ☹ | ☺ | ☺ | ☺ | 22 |
| Bruun | 1996 | Main | ☺ | ☹ | ☺ | ☹ | ☺ | ☺ | ☺ | ☺ | ☺ | ☺ | 26 |
| Chang | 1996 | Main | ☺ | ☺ | ☺ | ☹ | ☺ | ☺ | ☹ | ☺ | ☺ | ☺ | 26 |
| Denis | 1998 | Main | ☺ | ☹ | ☺ | ☺ | ☺ | ☺ | ☺ | ☺ | ☺ | ☺ | 28 |
| Iversen | 1993 | Main | ☺ | ☹ | ☹ | ☹ | ☹ | ☺ | ☺ | ☺ | ☺ | ☺ | 22 |
| Boccardo | 1993 | Main | ☺ | ☹ | ☺ | ☺ | ☺ | ☺ | ☹ | ☺ | ☺ | ☺ | 26 |
| De Voogt | 1998 | Main | ☺ | ☹ | ☹ | ☹ | ☹ | ☺ | ☹ | ☺ | ☺ | ☺ | 20 |
| Kaisary | 1991 | Main | ☺ | ☹ | ☺ | ☺ | ☺ | ☺ | ☹ | ☺ | ☺ | ☺ | 26 |
| Sharifi | 1985 | Main | ☺ | ☹ | ☹ | ☹ | ☹ | ☺ | ☹ | ☺ | ☺ | ☹ | 18 |
| Kotake | 1999 | Main | ☺ | ☹ | ☹ | ☺ | ☹ | ☺ | ☺ | ☺ | ☺ | ☺ | 30 |
| Lukkarinen | 1994 | Main | ☺ | ☹ | ☹ | ☹ | ☹ | ☺ | ☺ | ☺ | ☺ | ☹ | 20 |
| Organ | 2013 | Main | ☺ | ☹ | ☹ | ☺ | ☹ | ☺ | ☺ | ☺ | ☺ | ☺ | 24 |
| Crawford | 1989 | Main | ☺ | ☺ | ☹ | ☺ | ☹ | ☺ | ☺ | ☺ | ☺ | ☺ | 26 |
| Klotz | 2008 | Main | ☺ | ☹ | ☹ | ☹ | ☹ | ☺ | ☺ | ☺ | ☺ | ☺ | 22 |
| Akaza | 2003 | Main | ☺ | ☹ | ☹ | ☺ | ☹ | ☺ | ☹ | ☺ | ☺ | ☺ | 24 |
| Manikandan | 2005 | Main | ☺ | ☺ | ☹ | ☺ | ☹ | ☺ | ☹ | ☺ | ☺ | ☹ | 22 |
| Crook | 2009 | Main | ☺ | ☺ | ☺ | ☹ | ☺ | ☺ | ☺ | ☺ | ☺ | ☺ | 28 |
| Burns-Cox | 2002 | Main | ☺ | ☹ | ☹ | ☺ | ☹ | ☺ | ☹ | ☺ | ☺ | ☺ | 22 |
| Brisset | 1997 | Main | ☺ | ☺ | ☹ | ☺ | ☹ | ☺ | ☹ | ☺ | ☺ | ☺ | 24 |
| Mikkola | 1998 | Main | ☺ | ☹ | ☺ | ☹ | ☺ | ☺ | ☺ | ☺ | ☺ | ☹ | 24 |
| Vogelzang | 1995 | Main | ☺ | ☹ | ☺ | ☺ | ☹ | ☺ | ☹ | ☺ | ☺ | ☺ | 24 |
| Waymont | 1995 | Main | ☺ | ☹ | ☹ | ☺ | ☺ | ☺ | ☺ | ☺ | ☺ | ☺ | 26 |
| Thorpe | 1996 | Main | ☺ | ☹ | ☹ | ☺ | ☹ | ☺ | ☺ | ☺ | ☺ | ☺ | 24 |
| Robinson | 1995 | Main | ☺ | ☹ | ☹ | ☹ | ☹ | ☺ | ☺ | ☺ | ☺ | ☺ | 22 |
| Armstrong | 2011 | Main | ☺ | ☹ | ☺ | ☹ | ☺ | ☺ | ☺ | ☺ | ☺ | ☺ | 26 |
| Dijkman | 1997 | Main | ☺ | ☺ | ☹ | ☺ | ☹ | ☺ | ☹ | ☺ | ☺ | ☺ | 30 |
| Author | Year | Analysis | Was the assignment to treatment groups truly random? | Were participants blinded to treatment allocation? | Was allocation to treatment groups concealed from the allocator? | Were the outcomes of people who withdrew described and included in the analysis? | Were those assessing outcomes blind to the treatment allocation? | Were the control and treatment groups comparable at entry? | Were groups treated identically other than for the named interventions? | Were outcomes measured in the same way for all groups? | Were outcomes measured in a reliable way? | Was appropriate statistical analysis used? | Score final |
| Ostri | 1991 | Main | ☺ | ☹ | ☹ | ☺ | ☹ | ☺ | ☺ | ☺ | ☺ | ☹ | 22 |
| Wirth | 2004 | Main | ☺ | ☹ | ☹ | ☺ | ☹ | ☺ | ☹ | ☺ | ☺ | ☹ | 20 |
| Bales | 1996 | Main | ☺ | ☹ | ☹ | ☺ | ☹ | ☺ | ☺ | ☺ | ☺ | ☺ | 24 |
| Citrin | 1991 | Main | ☺ | ☹ | ☹ | ☺ | ☹ | ☺ | ☹ | ☺ | ☺ | ☺ | 22 |
| Ansari | 2004 | Main | ☺ | ☹ | ☹ | ☺ | ☹ | ☺ | ☺ | ☺ | ☺ | ☺ | 24 |
| Pavone-Macaluso | 1986 | Main | ☺ | ☺ | ☺ | ☺ | ☺ | ☺ | ☹ | ☺ | ☺ | ☺ | 28 |
| Parmar | 1988 | Main | ☺ | ☹ | ☹ | ☺ | ☹ | ☺ | ☹ | ☺ | ☺ | ☺ | 22 |
| Anderson | 2013 | Main | ☺ | ☹ | ☹ | ☺ | ☹ | ☺ | ☺ | ☺ | ☺ | ☹ | 22 |
| Tyrrell | 2000 | Main | ☺ | ☹ | ☹ | ☺ | ☹ | ☺ | ☺ | ☺ | ☺ | ☺ | 24 |
| Bono | 1998 | Main | ☺ | ☺ | ☺ | ☺ | ☺ | ☺ | ☺ | ☺ | ☺ | ☹ | 28 |
| Zalcberg | 1996 | Main | ☺ | ☺ | ☹ | ☺ | ☹ | ☺ | ☺ | ☺ | ☺ | ☺ | 26 |
| Aro | 1993 | Main | ☺ | ☺ | ☹ | ☺ | ☹ | ☺ | ☺ | ☺ | ☺ | ☹ | 24 |
| Navratil | 1987 | Main | ☺ | ☺ | ☹ | ☹ | ☹ | ☺ | ☺ | ☺ | ☺ | ☹ | 22 |
| Botto | 1989 | Main | ☺ | ☹ | ☹ | ☺ | ☹ | ☺ | ☺ | ☺ | ☺ | ☺ | 24 |

* when the treatment was adapted to the medical status of the patient (progression of prostate cancer, adverse effect…), we considered that the group were not identically treated.

Using the Johanna Briggs manual^1^.

1 The Joanna Briggs Institute, The University of Adelaide. Joanna Briggs Institute. Reviewer’s manual - 2011 Edition. 2011 http://joannabriggs.org/assets/docs/sumari/ReviewersManual-2011.pdf.

**Bibliography:**

- Akaza H, Homma Y, Okada K, et al. A prospective and randomized study of primary hormonal therapy for patients with localized or locally advanced prostate cancer unsuitable for radical prostatectomy: results of the 5-year follow-up. BJU Int. 2003;91(1):33-36.

- Akaza H, Hinotsu S, Usami M, et al. Combined androgen blockade with bicalutamide for advanced prostate cancer: Long-term follow-up of a phase 3, double-blind, randomized study for survival. Cancer. 2009;115(15):3437-3445.

- Anderson J, Al-Ali G, Wirth M, et al. Degarelix versus goserelin (+ antiandrogen flare protection) in the relief of lower urinary tract symptoms secondary to prostate cancer: results from a phase IIIb study (NCT00831233). Urol Int. 2013;90(3):321-328.

- Ansari MS, Gupta NP, Hemal AK, Dogra PN, Seth A. Combined androgen blockade in the management of advanced prostate cancer: a sensible or ostensible approach. Int J Urol Off J Jpn Urol Assoc. 2004;11(12):1092-1096.

- Armstrong JG, Gillham CM, Dunne MT, et al. A randomized trial (Irish clinical oncology research group 97-01) comparing short versus protracted neoadjuvant hormonal therapy before radiotherapy for localized prostate cancer. Int J Radiat Oncol Biol Phys. 2011;81(1):35-45.

- Aro J, Ruutu M, Juusela H, Hansson E, Permi J. Polyestradiol phosphate (160 mg/month) or LHRH analog (buserelin depot) in the treatment of locally advanced or metastasized prostatic cancer. The Finnprostate Group. Ann Chir Gynaecol Suppl. 1993;206:5-8.

- Bales GT, Chodak GW. A controlled trial of bicalutamide versus castration in patients with advanced prostate cancer. Urology. 1996;47(1A Suppl):38-43; discussion 48-53.

- Boccardo F, Pace M, Rubagotti A, et al. Goserelin acetate with or without flutamide in the treatment of patients with locally advanced or metastatic prostate cancer. Eur J Cancer. 1993;29(8):1088-1093.

- Boccardo F, Barichello M, Battaglia M, et al. Bicalutamide monotherapy versus flutamide plus goserelin in prostate cancer: updated results of a multicentric trial. Eur Urol. 2002;42(5):481-490.

- Bolla M, de Reijke TM, Van Tienhoven G, et al. Duration of androgen suppression in the treatment of prostate cancer. N Engl J Med. 2009;360(24):2516-2527..

- Bolla M, Van Tienhoven G, Warde P, et al. External irradiation with or without long-term androgen suppression for prostate cancer with high metastatic risk: 10-year results of an EORTC randomised study. Lancet Oncol. 2010;11(11):1066-1073.

- Bono AV, DiSilverio F, Robustelli della Cuna G, et al. Complete androgen blockade versus chemical castration in advanced prostatic cancer: analysis of an Italian multicentre study. Italian Leuprorelin Group. Urol Int. 1998;60 Suppl 1:18-24.

- Botto H, Richard F, Mathieu F, Camey M. Decapeptyl in the treatment of advanced prostatic cancer: comparative study with pulpectomy. Prog Clin Biol Res. 1989;303:53-60.

- Brisset JM, Boccon-Gibod L, Botto H, et al. Anandron (RU 23908) associated to surgical castration in previously untreated stage D prostate cancer: a multicenter comparative study of two doses of the drug and of a placebo. Prog Clin Biol Res. 1987;243 A:411-422

- Burns-Cox N, Basketter V, Higgins B, Holmes S. Prospective randomised trial comparing diethylstilboestrol and flutamide in the treatment of hormone relapsed prostate cancer. Int J Urol Off J Jpn Urol Assoc. 2002;9(8):431-434.

- Bruun E, Frimodt-Møller C. The effect of Buserelin versus conventional antiandrogenic treatment in patients with T2-4NXM1 prostatic cancer. A prospective, randomized multicentre phase III trial. The “Danish Buserelin Study Group.” Scand J Urol Nephrol. 1996;30(4):291-297.

- Calais da Silva FEC, Bono AV, Whelan P, et al. Intermittent androgen deprivation for locally advanced and metastatic prostate cancer: results from a randomised phase 3 study of the South European Uroncological Group. Eur Urol. 2009;55(6):1269-1277.

- Chang A, Yeap B, Davis T, et al. Double-blind, randomized study of primary hormonal treatment of stage D2 prostate carcinoma: flutamide versus diethylstilbestrol. J Clin Oncol Off J Am Soc Clin Oncol. 1996;14(8):2250-2257.

- Citrin DL, Resnick MI, Guinan P, et al. A comparison of Zoladex and DES in the treatment of advanced prostate cancer: results of a randomized, multicenter trial. The Prostate. 1991;18(2):139-146.

- Crawford ED, Eisenberger MA, McLeod DG, et al. A controlled trial of leuprolide with and without flutamide in prostatic carcinoma. N Engl J Med. 1989;321(7):419-424.

- Crook J, Ludgate C, Malone S, et al. Final report of multicenter Canadian Phase III randomized trial of 3 versus 8 months of neoadjuvant androgen deprivation therapy before conventional-dose radiotherapy for clinically localized prostate cancer. Int J Radiat Oncol Biol Phys. 2009;73(2):327-333.

- D’Amico AV, Chen M-H, Renshaw AA, Loffredo M, Kantoff PW. Androgen suppression and radiation vs radiation alone for prostate cancer: a randomized trial. JAMA. 2008;299(3):289-295.

- Denham JW, Steigler A, Lamb DS, et al. Short-term neoadjuvant androgen deprivation and radiotherapy for locally advanced prostate cancer: 10-year data from the TROG 96.01 randomised trial. Lancet Oncol. 2011;12(5):451-459.

- Denis LJ, Keuppens F, Smith PH, et al. Maximal androgen blockade: Final analysis of EORTC phase III trial 30853. Eur Urol. 1998;33(2):144-151.

- De Voogt HJ, Studer U, Schroder FH, Klijn JG, De Pauw M, Sylvester R. Maximum androgen blockade using LHRH agonist buserelin in combination with short-term (two weeks) or long-term (continuous) cyproterone acetate is not superior to standard androgen deprivation in the treatment of advanced prostate cancer. Final analysis of EORTC GU group trial 30843. Eur Urol. 1998;33(2):152-158.

- Dijkman GA, Janknegt RA, De Reijke TM, Debruyne FM. Long-term efficacy and safety of nilutamide plus castration in advanced prostate cancer, and the significance of early prostate specific antigen normalization. International Anandron Study Group. J Urol. 1997;158(1):160-163.

- Efstathiou JA, Bae K, Shipley WU, et al. Cardiovascular mortality and duration of androgen deprivation for locally advanced prostate cancer: analysis of RTOG 92-02. Eur Urol. 2008;54(4):816-823.

- Eisenberger MA, Blumenstein BA, Crawford ED, et al. Bilateral orchiectomy with or without flutamide for metastatic prostate cancer. N Engl J Med. 1998;339(15):1036-1042.

- Horwitz EM, Bae K, Hanks GE, et al. Ten-year follow-up of radiation therapy oncology group protocol 92-02: A phase III trial of the duration of elective androgen deprivation in locally advanced prostate cancer. J Clin Oncol. 2008;26(15):2497-2504.

- Hussain M, Tangen CM, Berry DL, et al. Intermittent versus continuous androgen deprivation in prostate cancer. N Engl J Med. 2013;368(14):1314-1325.

- Irani J, Celhay O, Hubert J, et al. Continuous versus six months a year maximal androgen blockade in the management of prostate cancer: a randomised study. Eur Urol. 2008;54(2):382-391.

- Iversen P, Rasmussen F, Klarskov P, Christensen IJ. Long-term results of Danish Prostatic Cancer Group trial 86. Goserelin acetate plus flutamide versus orchiectomy in advanced prostate cancer. *Cancer* 1993; **72**: 3851–4.

- Iversen P, McLeod DG, See WA, et al. Antiandrogen monotherapy in patients with localized or locally advanced prostate cancer: final results from the bicalutamide Early Prostate Cancer programme at a median follow-up of 9.7 years. BJU Int. 2010;105(8):1074-1081.

- Jones CU, Hunt D, McGowan DG, et al. Radiotherapy and short-term androgen deprivation for localized prostate cancer. N Engl J Med. 2011;365(2):107-118.

- Kaisary AV, Tyrrell CJ, Peeling WB, Griffiths K. Comparison of LHRH analogue (Zoladex) with orchiectomy in patients with metastatic prostatic carcinoma. Br J Urol. 1991;67(5):502-508.

- Klotz L, Boccon-Gibod L, Shore ND, et al. The efficacy and safety of degarelix: A 12-month, comparative, randomized, open-label, parallel-group phase III study in patients with prostate cancer. BJU Int. 2008;102(11):1531-1538.

- Kotake T, Usami M, Akaza H, et al. Goserelin acetate with or without antiandrogen or estrogen in the treatment of patients with advanced prostate cancer: A multicenter, randomized, controlled trial in Japan. Jpn J Clin Oncol. 1999;29(11):562-570.

- Lukkarinen O, Kontturi M. Comparison of a long-acting LHRH agonist and polyoestradiol phosphate in the treatment of advanced prostatic carcinoma. An open prospective, randomized multicentre study. Scand J Urol Nephrol. 1994;28(2):171-178.

- Manikandan R, Srirangam SJ, Pearson E, Brown SCW, O’Reilly P, Collins GN. Diethylstilboestrol versus bicalutamide in hormone refractory prostate carcinoma: a prospective randomized trial. Urol Int. 2005;75(3):217-221.

- Mikkola AK, Ruutu ML, Aro JL, Rannikko SA, Salo JO. Parenteral polyoestradiol phosphate vs orchidectomy in the treatment of advanced prostatic cancer. Efficacy and cardiovascular complications: a 2-year follow-up report of a national, prospective prostatic cancer study. Finnprostate Group. Br J Urol. 1998;82(1):63-68.

- Mottet N, Van Damme J, Loulidi S, et al. Intermittent hormonal therapy in the treatment of metastatic prostate cancer: a randomized trial. BJU Int. 2012;110(9):1262-1269.

- Navratil H. Double-blind study of Anandron versus placebo in stage D2 prostate cancer patients receiving buserelin. Results on 49 cases from a multicentre study. Prog Clin Biol Res. 1987;243A:401-410.

- Organ M, Wood L, Wilke D, et al. Intermittent LHRH therapy in the management of castrate-resistant prostate cancer (CRPCa): results of a multi-institutional randomized prospective clinical trial. Am J Clin Oncol. 2013;36(6):601-605.

- Ostri P, Bonnesen T, Nilsson T, Frimodt-Møller C. Treatment of symptomatic metastatic prostatic cancer with cyproterone acetate versus orchiectomy: a prospective randomized trial. Urol Int. 1991;46(2):167-171.

- Parmar H, Phillips RH, Lightman SL, Edwards L. How would you like to have an orchidectomy for advanced prostatic cancer? Am J Clin Oncol. 1988;11 Suppl 2:S160-S168.

- Pavone-Macaluso M, de Voogt HJ, Viggiano G, et al. Comparison of diethylstilbestrol, cyproterone acetate and medroxyprogesterone acetate in the treatment of advanced prostatic cancer: Final analysis of a randomized phase III trial of the European Organization for Research on Treatment of Cancer Urological Group. J Urol. 1986;136(3):624-631.

- Roach III M, Bae K, Speight J, et al. Short-term neoadjuvant androgen deprivation therapy and external-beam radiotherapy for locally advanced prostate cancer: Long-term results of RTOG 8610. J Clin Oncol. 2008;26(4):585-591.

- Robinson MR, Smith PH, Richards B, Newling DW, de Pauw M, Sylvester R. The final analysis of the EORTC Genito-Urinary Tract Cancer Co-Operative Group phase III clinical trial (protocol 30805) comparing orchidectomy, orchidectomy plus cyproterone acetate and low dose stilboestrol in the management of metastatic carcinoma of the prostate. Eur Urol. 1995;28(4):273-283.

- Sharifi R, Lee M, Ojeda L, Ray P, Stobnicki M, Guinan P. Comparison of leuprolide and diethylstilbestrol for stage D2 adenocarcinoma of prostate. Urology. 1985;26(2):117-124.

- Schröder FH, Whelan P, De Reijke TM, et al. Metastatic prostate cancer treated by Flutamide versus Cyproterone acetate: Final analysis of the “European Organization for Research and Treatment of Cancer” (EORTC) protocol 30892. Eur Urol. 2004;45(4):457-464.

- Smith MR, Klotz L, Persson B-E, Olesen TK, Wilde AAM. Cardiovascular safety of degarelix: results from a 12-month, comparative, randomized, open label, parallel group phase III trial in patients with prostate cancer. J Urol. 2010;184(6):2313-2319.

- Thorpe SC, Azmatullah S, Fellows GJ, Gingell JC, O’Boyle PJ. A prospective, randomised study to compare goserelin acetate (Zoladex(®)) versus cyproterone acetate (Cyprostat(®)) versus a combination of the two in the treatment of metastatic prostatic carcinoma. Eur Urol. 1996;29(1):47-54.

- Tyrrell CJ, Altwein JE, Klippel F, et al. Comparison of an LH-RH analogue (Goeserelin acetate, “Zoladex”) with combined androgen blockade in advanced prostate cancer: final survival results of an international multicentre randomized-trial. International Prostate Cancer Study Group. Eur Urol. 2000;37(2):205-211.

- Vogelzang NJ, Chodak GW, Soloway MS, et al. Goserelin versus orchiectomy in the treatment of advanced prostate cancer: final results of a randomized trial. Zoladex Prostate Study Group. Urology. 1995;46(2):220-226.

- Waymont B, Lynch TH, Dunn JA, et al. Phase III randomised study of zoladex versus stilboestrol in the treatment of advanced prostate cancer. Br J Urol. 1992;69(6):614-620

- Wirth MP, Weissbach L, Marx F-J, et al. Prospective randomized trial comparing flutamide as adjuvant treatment versus observation after radical prostatectomy for locally advanced, lymph node-negative prostate cancer. Eur Urol. 2004;45(3):267-270; discussion 270.

- Zalcberg JR, Raghaven D, Marshall V, Thompson PJ. Bilateral orchidectomy and flutamide versus orchidectomy alone in newly diagnosed patients with metastatic carcinoma of the prostate--an Australian multicentre trial. Br J Urol. 1996;77(6):865-869.

**Appendix eTable 7. Analysis from randomized controlled trials: the upper right side concern the indirect comparisons (network) with RR (95%CL) for overall death (the reference treatment appears in the column), and the lower left side concern the direct analysis (the reference treatment appears in the line).**

|  | AA | CAB continuous | CAB intermittent | CPT continuous | CPT intermittent | Estrogen | LHRH agonist | LHRH agonist + CMD long term | LHRH agonist + CMD short term | LHRH agonist + CPT | LHRH agonist + DES short term | LHRH antagonist | long term CAB | long term CMD | Medroxyprogesterone | OT | OT + AA | OT + CPT | Placebo | short term CAB |
| --- | --- | --- | --- | --- | --- | --- | --- | --- | --- | --- | --- | --- | --- | --- | --- | --- | --- | --- | --- | --- |
| AA | . | 1.23 [1.01-1.49] | 1.11 [0.83-1.48] | 0.87 [0.68-1.11] | 0.86 [0.57-1.29] | 1.13 [0.93-1.37] | 1.16 [0.98-1.37] | 1.08 [0.70-1.67] | 1.35 [0.86-2.13] | 1.16 [0.87-1.54] | 1.60 [1.00-2.58] | 2.26 [0.92-5.52] | 1.27 [0.92-1.77] | 1.03 [0.60-1.77] | 0.75 [0.53-1.09] | 1.11 [0.93-1.34] | 1.19 [0.93-1.52] | 1.10 [0.83-1.48] | 1.05 [0.91-1.21] | 1.11 [0.88-1.39] |
| CAB continuous | 1.07 [0.74-1.56] | . | 0.90 [0.73-1.12] | 0.71 [0.54-0.93] | 0.70 [0.45-1.07] | 0.92 [0.75-1.12] | 0.94 [0.82-1.08] | 0.88 [0.58-1.34] | 1.10 [0.71-1.72] | 0.94 [0.72-1.23] | 1.31 [0.82-2.08] | 1.84 [0.76-4.48] | 1.04 [0.73-1.48] | 0.84 [0.49-1.46] | 0.62 [0.42-0.90] | 0.91 [0.78-1.06] | 0.97 [0.78-1.21] | 0.90 [0.67-1.20] | 0.85 [0.70-1.03] | 0.90 [0.69-1.17] |
| CAB intermittent | x | 0.91 [0.81-1.02] | . | 0.78 [0.55-1.11] | 0.77 [0.48-1.25] | 1.02 [0.76-1.36] | 1.04 [0.81-1.34] | 0.98 [0.61-1.56] | 1.22 [0.74-1.99] | 1.04 [0.74-1.46] | 1.44 [0.87-2.41] | 2.03 [0.81-5.08] | 1.15 [0.76-1.73] | 0.93 [0.51-1.68] | 0.68 [0.44-1.05] | 1.00 [0.77-1.30] | 1.07 [0.79-1.46] | 0.99 [0.69-1.42] | 0.94 [0.71-1.26] | 1.00 [0.71-1.40] |
| CPT continuous | 0.82 [0.64-1.05] | x | x | . | 0.99 [0.71-1.38] | 1.30 [1.01-1.68] | 1.34 [1.03-1.72] | 1.25 [0.78-2.00] | 1.56 [0.95-2.56] | 1.33 [0.95-1.88] | 1.85 [1.11-2.09] | 2.61 [1.04-6.51] | 1.47 [1.00-2.18] | 1.19 [0.67-2.13] | 0.87 [0.63-1.22] | 1.29 [0.99-1.67] | 1.38 [1.01-1.87] | 1.27 [0.90-1.80] | 1.21 [0.93-1.57] | 1.28 [0.93-1.75] |
| CPT intermittent | x | x | x | 0.99 [0.80-1.23] | . | 1.32 [0.87-2.00] | 1.35 [0.89-2.05] | 1.26 [0.71-2.24] | 1.58 [0.87-2.86] | 1.35 [0.84-2.17] | 1.87 [1.02-3.44] | 2.63 [1.00-6.97] | 1.49 [0.89-2.48] | 1.21 [0.62-2.34] | 0.88 [0.55-1.41] | 1.30 [0.85-1.98] | 1.39 [0.89-2.18] | 1.29 [0.80-2.07] | 1.22 [0.80-1.86] | 1.29 [0.82-2.04] |
| Estrogen | 1.11 [0.86-1.42] | x | x | 1.09 [0.79-1.51] | x | . | 1.02 [0.86-1.22] | 0.96 [0.62-1.48] | 1.20 [0.76-1.89] | 1.02 [0.77-1.36] | 1.42 [0.88-2.29] | 2.00 [0.82-4.90] | 1.13 [0.79-1.61] | 0.92 [0.53-1.60] | 0.67 [0.48-0.95] | 0.99 [0.83-1.18] | 1.06 [0.83-1.34] | 0.98 [0.75-1.27] | 0.93 [0.76-1.14] | 0.98 [0.75-1.29] |
| LHRH agonist | x | 0.90 [0.82-1.00] | x | 1.39 [0.79-2.45] | x | 0.95 [0.81-1.13] | . | 0.94 [0.63-1.39] | 1.17 [0.77-1.79] | 1.00 [0.79-1.27] | 1.39 [0.89-2.16] | 1.95 [0.81-4.71] | 1.10 [0.79-1.54] | 0.89 [0.52-1.53] | 0.65 [0.46-0.94] | 0.96 [0.85-1.09] | 1.03 [0.84-1.27] | 0.95 [0.73-1.25] | 0.91 [0.78-1.06] | 0.96 [0.76-1.21] |
| LHRH agonist + CMD long term | x | x | x | x | x | x | x | . | 1.25 [0.82-1.90] | 1.07 [0.67-1.70] | 1.48 [0.96-2.30] | 2.09 [0.79-5.48] | 1.18 [0.70-1.98] | 0.95 [0.49-1.87] | 0.70 [0.41-1.20] | 1.03 [0.68-1.56] | 1.10 [0.70-1.72] | 1.02 [0.63-1.72] | 0.97 [0.63-1.48] | 1.02 [0.64-1.62] |
| LHRH agonist + CMD short term | x | x | x | x | x | x | 1.17 [0.69-1.65] | 1.25 [0.89-1.75] | . | 0.85 [0.52-1.39] | 1.19 [0.75-1.88] | 1.67 [0.63-4.43] | 0.94 [0.55-1.61] | 0.76 [0.38-1.52] | 0.56 [0.32-0.98] | 0.82 [0.53-1.28] | 0.88 [0.55-1.41] | 0.81 [0.49-1.35] | 0.77 [0.49-1.21] | 0.82 [0.50-1.33] |
| LHRH agonist + CPT | x | x | x | x | x | x | x | x | x | . | 1.39 [0.84-2.30] | 1.95 [0.78-4.87] | 1.10 [0.73-1.66] | 0.89 [0.50-1.61] | 0.66 [0.43-1.00] | 0.96 [0.76-1.23] | 1.03 [0.77-1.38] | 0.95 [0.67-1.35] | 0.91 [0.69-1.20] | 0.96 [0.69-1.34] |
| LHRH agonist + DES short term | x | x | x | x | x | x | 1.39 [0.96-2.00] | 1.48 [1.03-2.13] | 1.19 [0.80-1.75] | x | . | 1.41 [0.53-3.77] | 0.79 [0.46-1.38] | 0.64 [0.32-1.30] | 0.47 [0.27-0.84] | 0.69 [0.44-1.10] | 0.74 [0.46-1.21] | 0.69 [0.41-1.16] | 0.65 [0.41-1.01] | 0.69 [0.42-1.14] |
| LHRH antagonist | x | x | x | x | x | x | 1.82 [0.75-4.55] | x | x | x | x | . | 0.56 [0.22-1.44] | 0.46 [0.16-1.28] | 0.34 [0.13-0.87] | 0.49 [0.20-1.20] | 0.53 [0.21-1.30] | 0.49 [0.19-1.23] | 0.46 [0.19-1.13] | 0.49 [0.20-1.21] |
| long term CAB | x | x | x | x | x | x | x | x | x | x | x | x | . | 0.81 [0.45-1.47] | 0.59 [0.37-0.95] | 0.87 [0.62-1.24] | 0.94 [0.64-1.37] | 0.86 [0.57-1.31] | 0.82 [0.61-1.10] | 0.87 [0.66-1.14] |
| long term CMD | x | x | x | x | x | x | x | x | x | x | x | x | x | . | 0.73 [0.39-1.38] | 1.08 [0.62-1.87] | 1.15 [0.65-2.04] | 1.07 [0.59-1.94] | 1.01 [0.60-1.70] | 1.07 [0.62-1.85] |
|  | AA | CAB continuous | CAB intermittent | CPT continuous | CPT intermittent | Estrogen | LHRH agonist | LHRH agonist + CMD long term | LHRH agonist + CMD short term | LHRH agonist + CPT | LHRH agonist + DES short term | LHRH antagonist | long term CAB | long term CMD | Medroxyprogesterone | OT | OT + AA | OT + CPT | Placebo | short term CAB |
| Medroxyprogesterone | x | x | x | 1.24 [0.95-1.61] | x | 0.74 [0.55-0.99] | x | x | x | x | x | x | x | x | . | 1.47 [1.02-2.12] | 1.57 [1.06-2.34] | 1.46 [0.96-2.22] | 1.38 [0.96-2.00] | 1.46 [0.97-2.20] |
| OT | 1.76 [1.27-2.44] | 0.95 [0.85-1.06] | x | 1.49 [0.74-2.98] | x | 0.95 [0.83-1.09] | 0.93 [0.86-1.00] | x | x | 0.96 [0.88-1.06] | x | x | x | x | x | . | 1.07 [0.91-1.26] | 0.99 [0.76-1.28] | 0.94 [0.78-1.13] | 0.99 [0.77-1.28] |
| OT + AA | x | x | x | x | x | x | x | x | x | x | x | x | x | x | x | 1.11  [1.03-1.20] | . | 0.92 [0.68-1.25] | 0.88 [0.69-1.12] | 0.93 [0.69-1.26] |
| OT + CPT | x | x | x | x | x | 0.97 [0.85-1.11] | x | x | x | x | x | x | x | x | x | x | x | . | 0.95 [0.71-1.28] | 1.00 [0.71-1.42] |
| Placebo | 1.01 [0.93-1.09] | X | x | 1.00 [0.90-1.11] | x | x | 0.94 [0.84-1.05] | x | x | x | x | x | 0.63 [0.48-0.83] | 1.05 [0.81-1.36] | x | x | x | x | . | 1.06 [0.88-1.26] |
| short term CAB | x | x | x | x | x | x | x | x | x | x | x | x | 0.88 [0.74-1.05] | x | x | x | x | x | 0.99 [0.90-1.10] | . |

"AA": antiandrogen

"CAB": combined androgen blockade (LHRH antagonist + AA)

"CPT": cyproterone acetate

"LHRH": luteinizing hormone-releasing hormone = GnRH: gonadotropin-releasing hormone

"CMD": chlormadinone acetate

"DES": diethylstilbestrol

"OT": orchiectomy

- Short term CAB corresponded to 3 or 4 months treatment.

- Long term CAB to only 6 to 8 months treatment.

- Continuous treatment was a very long term (> 1 year or permanent) treatment contrary to intermittent treatment which was also given on a very long term but episodically often because of progression or relapse of prostate cancer disease.

- CMD long term: at least 24 months.

- CMD short term: 8 weeks.

- DES short term: 8 weeks.

**Appendix eTable 8. Net heat plot for all-cause mortality.**

The area of the gray squares displays the contribution of the direct estimate in design d (shown in the column) to the network estimate in design d’ (shown in the row). The colors are associated with the change in inconsistency between direct and indirect evidence in design d’ (shown in the row) after detaching the effect of design d (shown in the column). Blue colors indicate an increase and warm colors indicate a decrease (the stronger the intensity of the color, the stronger the change).

1: "AA"

3: "CAB continuous"

4: "CAB intermittent"

5: "CPT continuous"

6: "CPT intermittent"

8: "Estrogen"

9: "LHRH agonist"

10: "LHRH agonist + CMD long term"

11: "LHRH agonist + CMD short term"

12: "LHRH agonist + CPT"

13: "LHRH agonist + DES short term"

14: "LHRH agonist continuous"

15: "LHRH agonist intermittent"

16: "LHRH antagonist"

17: "long term CAB"

18: "long term CMD"

19: "Medroxyprogesterone"

20: "OT"

21: "OT + AA"

22: "OT + CPT"

23: "Placebo"

24 : "short term CAB"

**Appendix eText 1**

Medline query on the 28^th^ July 2014

(prostatic neoplasm[MeSH Terms] OR prostate neoplasm OR prostatic cancer OR prostate cancer) AND (androgen antagonists[MeSH Terms] OR androgen deprivation OR androgen suppression OR antiandrogen OR gonadotropin-releasing hormone[MeSH Terms] OR GnRH OR gonadotropin releasing hormone agonist OR gonadotropin releasing hormone antagonist OR luteinizing hormone releasing hormone agonist OR luteinizing hormone releasing hormone antagonist OR LHRH OR flutamide OR bicalutamide OR enzalutamide OR nilutamide OR leuprorelin OR leuprolide OR goserelin OR triptorelin OR buserelin OR histrelin OR degarelix OR cetrorelix OR abarelix OR cyproterone OR orchiectomy)

Article types

- Clinical Trial

- Clinical Trial, Phase I

- Clinical Trial, Phase II

- Clinical Trial, Phase III

- Clinical Trial, Phase IV

- Comparative Study

- Congresses

- Controlled Clinical Trial

- Multicenter Study

- Pragmatic Clinical Trial

- Randomized Controlled Trial

- Observational study

Species

Humans

**Appendix eText 2. Script network meta-analysis for overall death**

#ouverture des librairies

library (rmeta);library(meta); library (netmeta);library(base)

setwd("C:/Users/florian/Desktop")

getwd ()

# on commence par la description des études

tab1<-read.csv2 ("ANTI_A.csv", header=T)

tab2<-subset(tab1, tab1$ANALYSE == "OK")

tab2$Treat_1 <- factor(tab2$Treat_1)

summary(tab2$Treat_1)

tab2$Treat_2<-factor(tab2$Treat_2)

summary(tab2$Treat_2)

netmetaresults <- netmeta(TE=TE,seTE=se.TE, treat1=Treat_1, treat2=Treat_2, studlab=author.study, data=tab2, sm="OR")

summary(netmetaresults)

# Clairement, utiliser le modèle à effet aléatoire

netmetaresults2 <- netmeta(TE=TE,seTE=se.TE, treat1=Treat_1, treat2=Treat_2, studlab=author.study, data=tab2, comb.fixed=FALSE, comb.random=TRUE, sm="OR")

summary(netmetaresults2)

# FAIT LE GRAPHE

netgraph(netmetaresults2,multiarm=FALSE)

netgraph(netmetaresults2)

# COMPARAISONS VERSUS PLACEBO

summary(netmetaresults2,ref="PLACEBO",digits=2)

# Forest plot vs PLACEBO

forest(netmetaresults2,ref="Placebo")

# Assess the degree of heterogeneity and inconsistency

# Heterogeneity and inconsistency statistics.

netmetaresults2$Q

netmetaresults2$df

netmetaresults2$pval.Q

netmetaresults2$Q.heterogeneity

netmetaresults2$Q.inconsistency

netmetaresults2$Q.decomp

netmetaresults2$I2

print(netmetaresults2)

# via decomposition

decomp.design(netmetaresults2)

# quantifying proportion of direct evidence, mean path length, minimal parallelism.

netmeasures(netmetaresults2)

# On renomme avec des petits noms (chiffres) pour faire un netheat plot ;

import_dataset<-read.csv2 ("import_dataset.csv", header=T)

alltreatments <- sort(unique(import_dataset[,"treatment"]))

# On renomme pour le traitement 1

tab2$T1N[tab2$Treat_1=="AA"]<-1

tab2$T1N[tab2$Treat_1=="ABIRA"]<-2

tab2$T1N[tab2$Treat_1=="CAB continuous"]<-3

tab2$T1N[tab2$Treat_1=="CAB intermittent"]<-4

tab2$T1N[tab2$Treat_1=="CPT continuous"]<-5

tab2$T1N[tab2$Treat_1=="CPT intermittent"]<-6

tab2$T1N[tab2$Treat_1=="ENZ"]<-7

tab2$T1N[tab2$Treat_1=="Estrogen"]<-8

tab2$T1N[tab2$Treat_1=="LHRH agonist"]<-9

tab2$T1N[tab2$Treat_1=="LHRH agonist + CMD long term"]<-10

tab2$T1N[tab2$Treat_1=="LHRH agonist + CMD short term"]<-11

tab2$T1N[tab2$Treat_1=="LHRH agonist + CPT"]<-12

tab2$T1N[tab2$Treat_1=="LHRH agonist + DES short term"]<-13

tab2$T1N[tab2$Treat_1=="LHRH agonist continuous"]<-14

tab2$T1N[tab2$Treat_1=="LHRH agonist intermittent"]<-15

tab2$T1N[tab2$Treat_1=="LHRH antagonist"]<-16

tab2$T1N[tab2$Treat_1=="long term CAB"]<-17

tab2$T1N[tab2$Treat_1=="long term CMD"]<-18

tab2$T1N[tab2$Treat_1=="Medroxyprogesterone"]<-19

tab2$T1N[tab2$Treat_1=="OT"]<-20

tab2$T1N[tab2$Treat_1=="OT + AA"]<-21

tab2$T1N[tab2$Treat_1=="OT + CPT"]<-22

tab2$T1N[tab2$Treat_1=="Placebo"]<-23

tab2$T1N[tab2$Treat_1=="short term CAB"]<-24

tab2$T1N

# On renomme pour le traitement 2

tab2$T2N[tab2$Treat_2=="AA"]<-1

tab2$T2N[tab2$Treat_2=="ABIRA"]<-2

tab2$T2N[tab2$Treat_2=="CAB continuous"]<-3

tab2$T2N[tab2$Treat_2=="CAB intermittent"]<-4

tab2$T2N[tab2$Treat_2=="CPT continuous"]<-5

tab2$T2N[tab2$Treat_2=="CPT intermittent"]<-6

tab2$T2N[tab2$Treat_2=="ENZ"]<-7

tab2$T2N[tab2$Treat_2=="Estrogen"]<-8

tab2$T2N[tab2$Treat_2=="LHRH agonist"]<-9

tab2$T2N[tab2$Treat_2=="LHRH agonist + CMD long term"]<-10

tab2$T2N[tab2$Treat_2=="LHRH agonist + CMD short term"]<-11

tab2$T2N[tab2$Treat_2=="LHRH agonist + CPT"]<-12

tab2$T2N[tab2$Treat_2=="LHRH agonist + DES short term"]<-13

tab2$T2N[tab2$Treat_2=="LHRH agonist continuous"]<-14

tab2$T2N[tab2$Treat_2=="LHRH agonist intermittent"]<-15

tab2$T2N[tab2$Treat_2=="LHRH antagonist"]<-16

tab2$T2N[tab2$Treat_2=="long term CAB"]<-17

tab2$T2N[tab2$Treat_2=="long term CMD"]<-18

tab2$T2N[tab2$Treat_2=="Medroxyprogesterone"]<-19

tab2$T2N[tab2$Treat_2=="OT"]<-20

tab2$T2N[tab2$Treat_2=="OT + AA"]<-21

tab2$T2N[tab2$Treat_2=="OT + CPT"]<-22

tab2$T2N[tab2$Treat_2=="Placebo"]<-23

tab2$T2N[tab2$Treat_2=="short term CAB"]<-24

tab2$T2N

netmetaresults3 <- netmeta(TE=TE,seTE=se.TE, treat1=T1N, treat2=T2N, studlab=author.study, data=tab2, comb.fixed=FALSE, comb.random=TRUE, sm="OR", reference="17")

summary(netmetaresults3)

# Visual inspection via netheat plot.

netheat(netmetaresults3)
